# Gut-derived acetate promotes B10 cells with antiinflammatory effects

**DOI:** 10.1172/jci.insight.144156

**Published:** 2021-04-08

**Authors:** C.I. Daïen, J. Tan, R. Audo, J. Mielle, L.E. Quek, J.R. Krycer, A. Angelatos, M. Duraes, G. Pinget, D. Ni, R. Robert, M.J. Alam, M.C.B. Amian, F. Sierro, A. Parmar, G. Perkins, S. Hoque, A.K. Gosby, S.J. Simpson, R.V. Ribeiro, C.R. Mackay, L. Macia

**Affiliations:** 1 Charles Perkins Centre, The University of Sydney, New South Wales, Sydney, Australia.; 2 Faculty of Medicine and Health, The University of Sydney School of Medicine, New South Wales, Sydney, Australia.; 3 Department of Rheumatology, Montpellier Hospital, University of Montpellier, Montpellier, France.; 4 Institute of Molecular Genetics of Montpellier, UMR5535, University of Montpellier, Montpellier, France.; 5 Human Health, Nuclear Science & Technology and Landmark Infrastructure (NSTLI) Australian Nuclear Science and Technology Organisation, New South Wales, Sydney, Australia.; 6 School of Mathematics and Statistics and; 7 School of Life and Environmental Sciences, The University of Sydney, New South Wales, Sydney, Australia.; 8 Department of Gynecology, Montpellier Hospital, University of Montpellier, Montpellier, France.; 9 Department of Physiology and; 10 Department of Microbiology, Biomedicine Discovery Institute, Monash University, Clayton, Victoria, Australia.; 11 Brain and Mind Centre, The University of Sydney, New South Wales, Sydney, Australia.; 12 Biosciences platform, NSTLI Australian Nuclear Science and Technology Organisation, New South Wales, Sydney, Australia.

**Keywords:** Immunology, Metabolism, Beta cells

## Abstract

Autoimmune diseases are characterized by a breakdown of immune tolerance partly due to environmental factors. The short-chain fatty acid acetate, derived mostly from gut microbial fermentation of dietary fiber, promotes antiinflammatory Tregs and protects mice from type 1 diabetes, colitis, and allergies. Here, we show that the effects of acetate extend to another important immune subset involved in tolerance, the IL-10–producing regulatory B cells (B10 cells). Acetate directly promoted B10 cell differentiation from mouse B1a cells both in vivo and in vitro. These effects were linked to metabolic changes through the increased production of acetyl-coenzyme A, which fueled the TCA cycle and promoted posttranslational lysine acetylation. Acetate also promoted B10 cells from human blood cells through similar mechanisms. Finally, we identified that dietary fiber supplementation in healthy individuals was associated with increased blood-derived B10 cells. Direct delivery of acetate or indirect delivery via diets or bacteria that produce acetate might be a promising approach to restore B10 cells in noncommunicable diseases.

## Graphical abstract

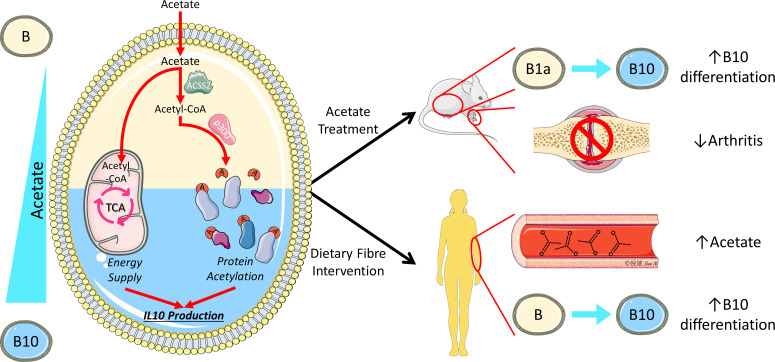

## Introduction

Debilitating autoimmune diseases such as rheumatoid arthritis can arise from the breakdown of immune tolerance. The cause of such breakdown is not fully known but a key feature is the expansion of proinflammatory Th17 and Th1 T cell subsets, with the concomitant decrease of antiinflammatory Treg subset (1). A minor subset of B cells, which releases the antiinflammatory cytokine IL-10, also plays an important role in immune tolerance. These regulatory B cells, also called B10 cells, promote Treg differentiation while inhibiting Th1 and Th17 cells (2). The key role of B10 cells in tolerance has been established in both preclinical and clinical studies. Indeed, the absence of B10 cells in mice exacerbates the development of arthritis (3), whereas adoptive transfer of B10 cells significantly decreases autoimmune disease severity in mouse models of experimental autoimmune encephalitis (4), colitis (5), and arthritis (6). Patients with rheumatoid arthritis are characterized by a significantly decreased proportion of B10 cells with impaired tolerogenic function (7). The proportion of B10 cells in these patients is also inversely correlated with the disease activity (7). Restoring both the function and the proportion of B10 cells in these patients could thus serve as a new therapeutic approach that may be applicable to other inflammatory diseases.

In mice, B10 cells mostly originate from peritoneal progenitors, B1a cells. B10 cells are also found to a lesser extent in the spleen, derived from B2 progenitor B cells, especially T2-marginal zone precursors (8). In humans, blood-derived B10 cells mainly derive from CD24^hi^CD38^hi^ and CD24^hi^CD27^+^ B cells (9). B10 cell differentiation is driven by environmental cues, with proinflammatory cytokines such as IL-21 having promoting effects (2). Interestingly, microorganisms can also directly potentiate B10 cell differentiation through binding of microbe-associated molecular patterns to TLRs. Bacterial DNA CpG motif is the most potent inducer of B10 cells via TLR9 activation (10). Gut bacteria can also indirectly promote B10 cell development through the induction of IL-6 and IL-1β (11), suggesting that commensal bacteria can promote B10 cell development independently of TLR activation.

Advances in the field of host interaction with its gut microbiota have highlighted that metabolites released by gut bacteria play a key role in immune cell differentiation and function (12). The short-chain fatty acids (SCFAs) acetate, butyrate, and propionate are released by gut bacterial fermentation of complex carbohydrates in the colon (13). We and others have shown that SCFAs are central players in immune tolerance by promoting Treg development either directly (14, 15), or indirectly through the activation of tolerogenic CD103^+^ dendritic cells in mice (16). Acetate also affects Treg development via G protein–coupled receptor (GPCR) activation, particularly GPR43 (12, 16) or histone deacetylase inhibition (15). Furthermore, acetate influences cell metabolism, particularly lipid and carbohydrate metabolism, by its conversion into acetyl coenzyme A (acetyl-CoA) by acetyl-CoA synthetase (ACSS) and subsequent entry into the TCA cycle. As such, acetate has been shown to promote CD8^+^ memory T cells as well as plasma B cell isotype switching by modulating TCA cycle activity, fatty-acid synthesis, and glycolysis (17, 18). Cytoplasmic acetyl-CoA can also result from the conversion of the TCA cycle intermediate citrate by ATP citrate lyase (ACLY). Acetyl-CoA is also a substrate for lysine acetylation, a posttranslational modification that regulates key cellular processes, including energy metabolism (19). Lysine-acetylation of glyceraldehyde 3-phosphate dehydrogenase in CD8^+^ T cells in the presence of acetate increased glycolysis-promoting naive CD8^+^ T cell differentiation into memory T cells (18). Together, SCFAs are emerging as key regulators of the immune response, potentially acting through numerous mechanisms to induce immune tolerance. A recent study has shown an indirect effect of butyrate on B10 cell development through the gut bacterial production of serotonin-derived metabolite 5-hydroxyindole-3-acetic acid (20). Another study has shown that the SCFA acetate potentiated the effect of CpG and LPS on B10 cell induction (21). Yet, whether SCFAs could directly affect the differentiation of B10 cells is unknown.

In the present work, we demonstrate that acetate directly promoted the differentiation of B10 cells in vivo and in vitro in both mice and humans. Acetate-promoted B10 cells were immunosuppressive in vivo, decreasing collagen antibody induced arthritis severity when adoptively transferred. Although the human peritoneal cavity was a poor reservoir of B10 cell progenitors, acetate promoted the differentiation of B10 cells from human PBMCs. Acetate induced functional B10 cells, which effectively promoted the differentiation of naive T cells into Tregs. Short-term dietary fiber intervention in women led to increased plasma acetate and proportion of B10 cells, showing that acetate was a potent inducer of B10 cells in humans. By using specific inhibitors, we identified that acetate induced B10 cell differentiation through its conversion into acetyl-CoA. This fueled the TCA cycle and increased protein acetylation. This work highlights the therapeutic potential for the SCFA acetate by inducing B10 cells to restore immune tolerance. This suggests the exciting possibility that our gut microbiome, and by extension our diet, could be utilized to treat inflammatory diseases.

## Results

### Acetate promoted the differentiation of IL-10–producing B cells from mouse B1a precursors in vitro.

SCFAs can directly induce Treg development (22, 14), but whether this effect applies to B10 cells is unknown. We confirmed that B10 cells, defined in our study as IL-10^+^–producing CD19^+^ B cells by flow cytometry (gating strategy indicated in Supplemental Figure 1A; supplemental material available online with this article; https://doi.org/10.1172/jci.insight.144156DS1), were enriched in mouse peritoneal cavity and to a lesser extent in the spleen, but were poorly represented in colon, mesenteric, and inguinal lymph nodes after TLR9 stimulation by CpG (Supplemental Figure 1B). We thus focused on peritoneal cells to study B10 cells.

**Figure 1:**
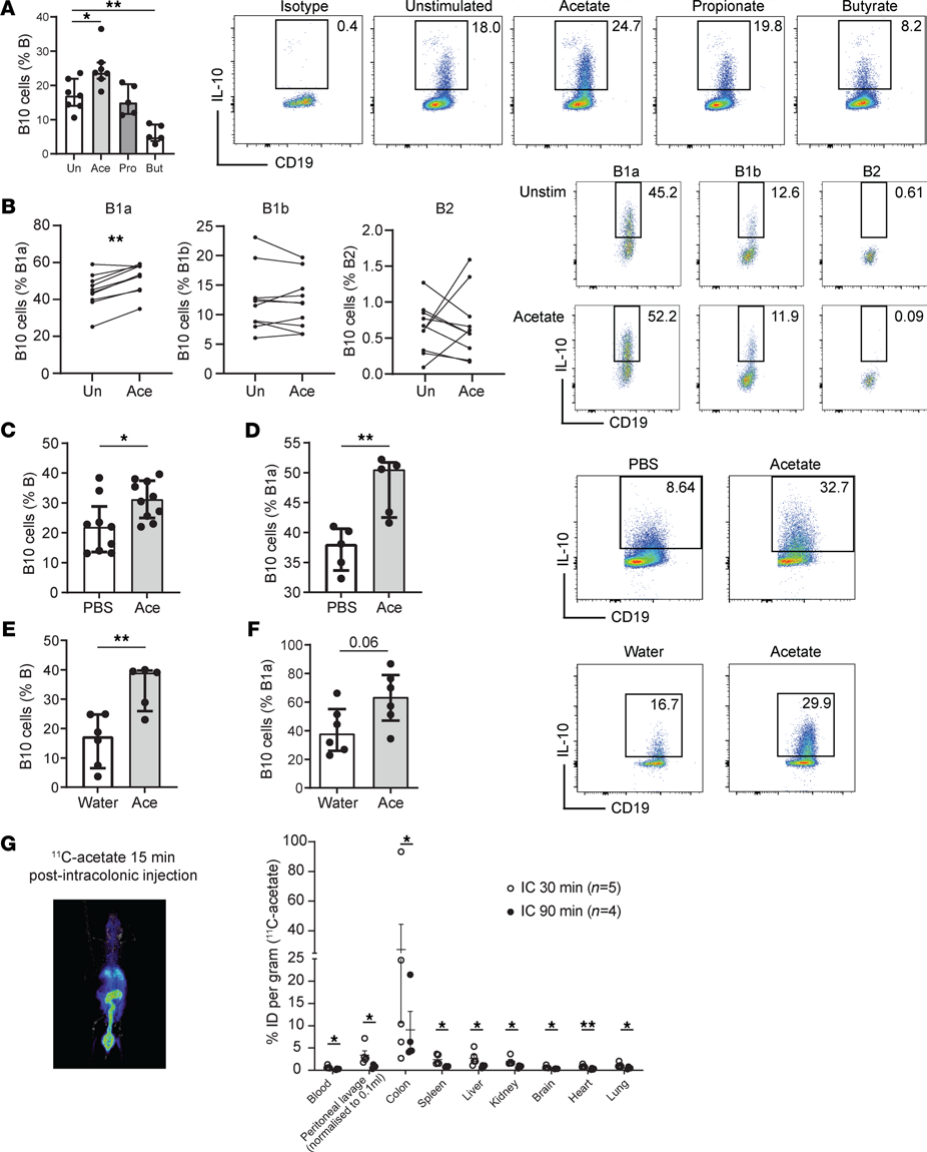
Acetate promotes the differentiation of IL-10–producing B cells from mouse B1a precursors. (**A**) 10^6^ peritoneal cells (*n* = 6–8) were incubated overnight with acetate (10 mM), butyrate (1 mM), or propionate (1 mM) and B10 cells (IL-10^+^ of CD19^+^ cells) characterized by flow cytometry. (**B**) 10^6^ peritoneal cells (*n* = 8) were incubated overnight with acetate (10 mM) and B10 cells among B1a (CD19^+^CD5^+^CD23^–^), B1b (CD19^+^CD5^–^CD23^–^), and B2 (CD19^+^CD5^–^CD23^+^) cells quantified by flow cytometry. The proportion of peritoneal B10 cells among B cells (**C**) (*n* = 9) or among B1a cells (**D**) (*n* = 5) was determined from C57BL/6 male mice injected i.p. twice at a 12-hour interval with acetate (500 mg/kg, pH-adjusted, *n* = 10) or PBS (*n* = 9). The proportion of peritoneal B10 cells among B cells (**E**) or among B1a cells (**F**) was determined from cells isolated from *n* = 6 mice treated with 200 mM acetate in drinking water for 3 weeks. Results were confirmed in 3 to 4 independent experiments. (**G**) PET scan image 15 minutes after intracolonic administration of ^11^C-acetate (left-hand side of panel) and biodistribution of ^11^C-acetate quantified as intensity dose per gram (%ID per gram) after 30- or 90-minute administration (right-hand side of panel). Intensity of 11C-acetate in the colon was significantly higher than all other studied organs both after 30- and 90-minute administration (*P* < 0.05 by ANOVA). One-way ANOVA Kruskal-Wallis tests for multiple comparisons were performed and Mann-Whitney *U* tests for 2-group comparison. Median (IQR) are represented. *P < 0.05; **P < 0.01. Un, unstimulated.

To determine the direct effect of SCFAs on mouse B10 cell induction, peritoneal cells were cultured overnight with acetate, butyrate, or propionate at doses previously reported for in vitro experiments (23, 24). Acetate significantly increased the proportion of B10 cells among peritoneal B cells (Figure 1A). On the other hand, propionate had no significant effect, whereas butyrate had inhibitory effects (Figure 1A). Interestingly, B10 cells were induced during overnight culture in the absence of stimulation, which was further increased by acetate (Supplemental Figure 1C). Next, we determined whether acetate promoted B10 cells equally in all subsets of B cells. By refining our gating strategy (Supplemental Figure 1D), we identified that acetate significantly induced IL-10 production in CD19^+^CD5^+^CD23^–^ B1a and not in CD19^+^CD5^–^CD23^–^ B1b or CD19^+^CD5^–^CD23^+^ B2 B cells (Figure 1B). This result is consistent with the low proportion of B10 cells observed in the spleen (Supplemental Figure 1B), which contains more conventional B2 B cells than B1 cells compared with the peritoneal cavity (Supplemental Figure 1E). Furthermore, acetate did not induce IL-10 production in marginal zone B cells, type 2 marginal zone B cells, plasma cells, or Tim-1–expressing B cells (Supplemental Figure 1F). Finally, acetate did not induce the proliferation of existing B10 cells because no changes in BrdU incorporation were observed (Supplemental Figure 1G), nor did it affect cell viability (Supplemental Figure 1H). Thus, direct induction of IL-10–producing B10 cells by acetate acted on B1a progenitors and was independent of cell proliferation and survival.

### Acetate promoted IL-10–producing B cells in vivo in mice.

To determine whether a local increase of acetate in the peritoneal cavity might promote B10 cells in vivo, we injected mice twice i.p. with pH-adjusted acetate at 500 mg/kg at 12-hour intervals. We identified that acetate treatment increased peritoneal B10 cell differentiation (Figure 1, C and D) without affecting the total proportion of B1a cells (Supplemental Figure 1I). This result suggests that a local rise of acetate in the peritoneum increased B10 cells in vivo. We and others have shown that the administration of acetate in drinking water could increase the proportion of Tregs in vivo via various mechanisms (15, 16, 22). To determine whether this effect was also observed with B10 cells, we administered acetate in drinking water to mice for 3 weeks. Although acetate did not affect the proportion of total B1a cells in the peritoneal cavity (Supplemental Figure 1J), it promoted B10 cell differentiation from peritoneal total B cells and from those with B1a cell phenotype (Figure 1, E and F). Consistent with our in vitro data (Figure 1A), the addition of propionate to drinking water did not induce B10 cells, whereas butyrate had inhibitory effects (data not shown). To validate that gut-derived acetate could reach the peritoneal cavity, we performed a biodistribution analysis of ^11^C-acetate after its intracolonic administration. As excepted, the intensity of ^11^C-acetate dose per gram of tissue (%ID per gram) was significantly highest in the colon, followed by the peritoneal cavity 30 minutes after administration, though it was mostly cleared after 90 minutes (Figure 1G). Intravenous administration of ^11^C-acetate, used as a positive control, resulted in systemic distribution of acetate (Supplemental Figure 1K).

Together, these results show that gut-derived acetate reached the peritoneal cavity and that both chronic and acute administration of acetate promoted B10 cell differentiation in vivo.

### IL-10–producing B cells induced by acetate were functional and decreased arthritis severity when adoptively transferred.

**Figure 2:**
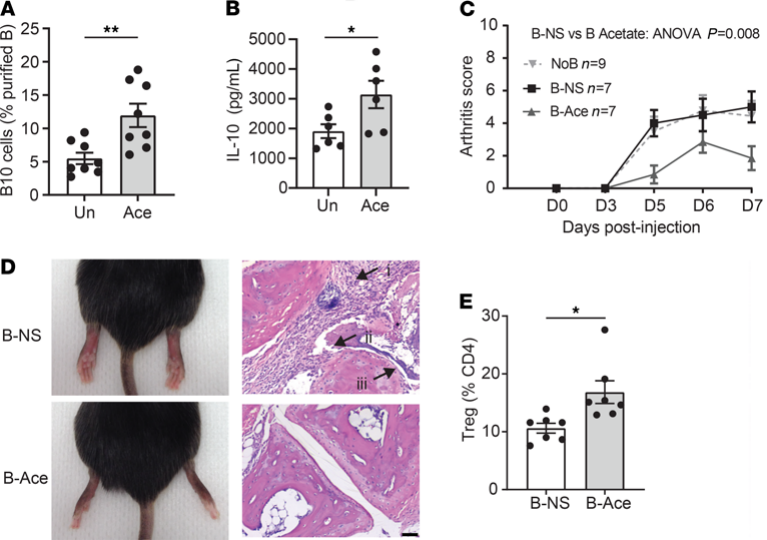
Acetate-induced B10 cells are functional in vitro and in vivo. (**A**) The proportion of IL-10^+^ cells was assessed among purified peritoneal B cells by flow cytometry. Cells were either unstimulated or incubated overnight with 10 mM acetate (Ace) (from *n* = 8 mice per group). (**B**) IL-10 quantification by ELISA in supernatants of purified B cells incubated overnight with 10 mM acetate (*n* = 6 per group). (**C**) Collagen antibody induced arthritis was induced by injecting i.p 4 mg anticollagen monoclonal antibodies on day 0 and 50 μg LPS on day 3. 10^6^ of nonstimulated (B-NS) or overnight stimulated peritoneal B cells with 10 mM acetate only (B-Ace) were injected i.p 2 hours after anticollagen antibody injection. Joints were monitored for 7 days and (**D**) collected for histological analysis (*n* = 7–9 per group). Increased cellular infiltrated, hyperplasia, and pannus formation (indicated by i–iii, respectively) were seen in B-NS groups compared with B-Ace groups (scale bar: 100 μm). (**E**) The proportion of splenic CD4^+^CD25^+^FoxP3^+^ Tregs was assessed by flow cytometry. Means and SEM are presented. Two-way ANOVA was performed and Wilcoxon paired tests for 2-group comparisons with *P < 0.05 and **P < 0.01. Un, unstimulated.

The peritoneal cavity is populated by a diverse range of immune cells such as macrophages and T cells, which could potentially affect B10 cell differentiation through their release of cytokines. To determine whether acetate promoted B10 cell development via indirect or direct effects, we incubated purified peritoneal B cells with acetate. We found that acetate induced B10 cells from isolated B cells by flow cytometry (Figure 2A) as well as their release of IL-10 by ELISA (Figure 2B). Thus, acetate directly promoted B10 cell differentiation.

IL-10 has been shown to control the severity of the inflammatory model of collagen autoantibody–induced-arthritis (CAIA), with IL-10–deficient mice exhibiting more severe disease in this model (25). We thus hypothesized that the transfer of IL-10–producing B10 cells would attenuate CAIA. To test this, mice were injected i.p. with 10^6^ B cells treated with either acetate or PBS, 2 hours after disease induction. Compared with mice injected with PBS-stimulated B cells (Figure 2C), mice treated with acetate-stimulated B cells had a significantly decreased disease development, better histopathological outcomes (Figure 2D), and an increased proportion of splenic Tregs (Figure 2E), confirming that B10 cells could decrease CAIA disease severity and that acetate induced functional B10 cells. Of note, we also found that the treatment with acetate had similar effects with mice that received acetate in drinking water for 3 weeks before and throughout the CAIA disease model: they had significantly decreased arthritis severity, better histological outcomes, and increased splenic Tregs compared with untreated mice (Supplemental Figure 2, A–C). Altogether, these data show that acetate promoted functional B10 cells that efficiently protects from arthritis development.

### Acetate promoted i.p. IL-10–producing B cells through metabolic changes and protein acetylation.

Acetate can promote Treg development by signaling either through specific GPCRs or epigenetic changes (15, 22). GPR43, GPR41, and GPR109a are the main GPCRs for SCFAs (13). Although GPR109 does not bind acetate (26) and GPR41 is not expressed by B cells (17, 27), GPR43 is thus the only relevant candidate receptor to study the impact of acetate on B10 cells. To determine whether acetate induced B10 cells through the activation of GPR43, we incubated i.p. *Gpr43*^–/–^ B cells with acetate. Acetate still significantly induced B10 cell differentiation in the absence of GPR43, suggesting that this receptor was not necessary (Supplemental Figure 3A). We further confirmed that the expression of GPR40, GPR41, and GPR43 in B1 cells was low by using open source RNA-Seq data set (28) (Supplemental Figure 3B), which is aligned with previous reports showing that B cells do not express receptors for SCFAs (29). Acetate can also modulate gene expression through histone acetylation, with increased histone acetylation at the Foxp3 promoter region promoting Treg development (15). To determine whether acetate increases IL-10 expression through this mechanism, we quantified histone H3 acetylation at the IL-10 promoter region via chromatin immunoprecipitation assay on acetate-treated B1a cells. We identified that the treatment with acetate did not affect histone acetylation in either the proximal or the distal promoter region of the IL-10 gene (Supplemental Figure 3, C and D). Thus, acetate promoted IL-10 production in B cells via mechanisms independent of GPCR activation or epigenetic changes in IL-10 promoter.

**Figure 3:**
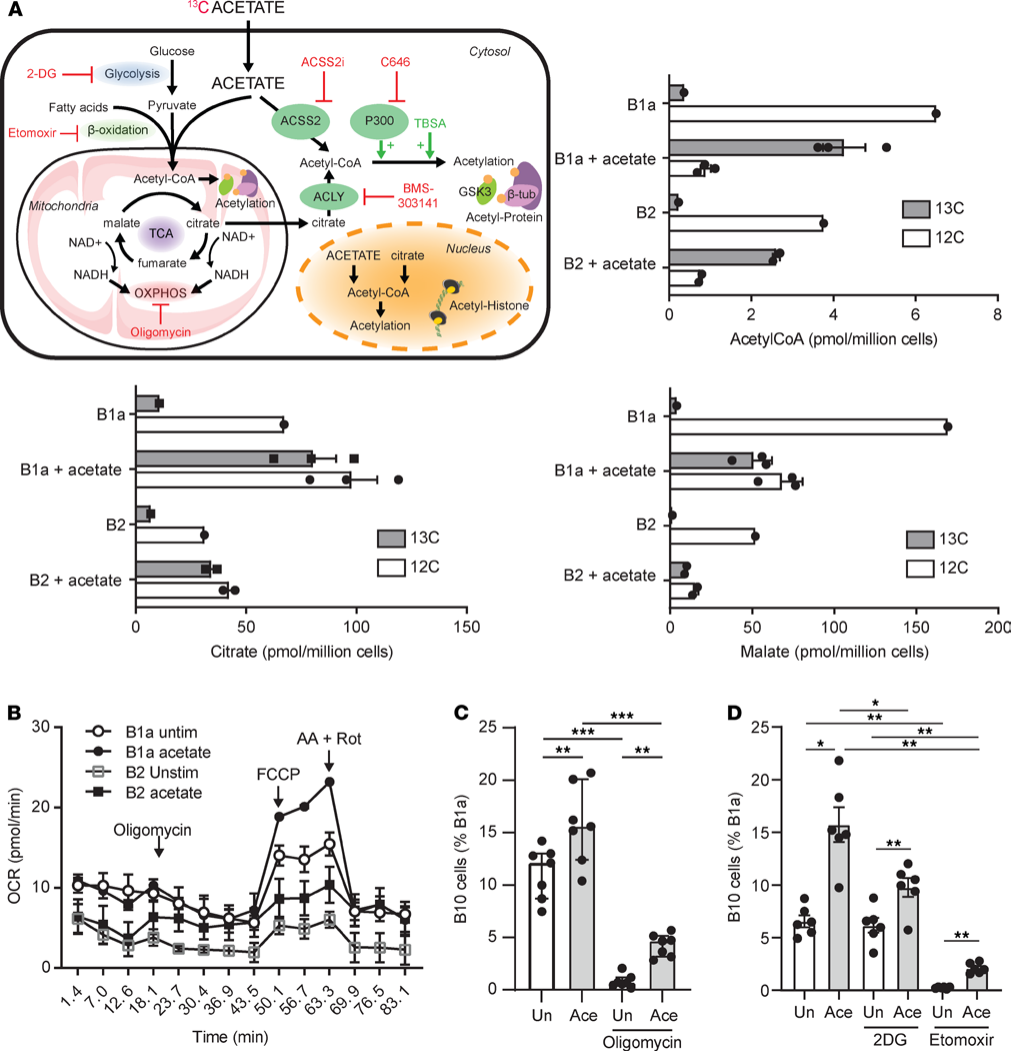
Acetate promotes B10 cells independently of GPR43 and HDAC inhibition but through metabolic changes. (**A**) To determine whether acetate affected B1a cells metabolism, we incubated sorted 10^6^ B1a cells (CD19^+^CD5^+^CD23^–^) or B2 cells (CD19^+^CD5^–^CD23^+^) from *n* = 20 pooled mice for 6 hours with ^13^C acetate alone and analyzed changes in metabolites as well as incorporation of ^13^C into acetyl-CoA, citrate and malate by liquid chromatography–mass spectrometry. (**B**) Real-time OCR (pmol/min) was measured by seahorse from 0.5 × 10^6^ sorted B1a or B2 cells stimulated overnight with or without acetate (10 mM) at baseline and after sequential injection of oligomycin (2 μM), FCCP (1 μM), and Antimycin A plus Rotenone (1 μM each). *P* < 0.0001 for time x group interaction by 2-way ANOVA and mean (SEM) are presented. 10^6^ peritoneal cells were incubated overnight with 1 μM oligomycin (**C**), 1 mM 2-DG, or 200 μM etomoxir (**D**) in the presence or absence of 10 mM acetate; proportion of B10 cells among B1a cells quantified by flow cytometry (*n* = 6–8). Results are represented as median (IQR) with *P < 0.05, **P < 0.01, and ***P < 0.005 by Mann Whitney *U* or 1 way-ANOVA. Results were confirmed in 2 to 3 independent experiments. HDAC, histone deacetylase; OCR, oxygen consumption rate; Un, unstimulated.

Apart from these mechanisms, acetate can be converted into acetyl-CoA, which fuels the TCA cycle and is an important acetyl group donor for protein acetylation. To follow the fate of acetate, we incubated purified B1a cells or B2 cells for 6 hours with ^13^C acetate and analyzed changes in metabolites by liquid chromatography–coupled mass spectrometry (LC-MS). Acetyl-CoA exhibited a high enrichment of acetate carbon in both cell subsets (Figure 3A), demonstrating the entry of acetate in both cell subsets. Relative incorporation of ^13^C into acetyl-CoA and TCA intermediates was similar in both cell subsets (Supplemental Figure 3E), but the absolute quantity of citrate, malate, and fumarate in B1a cells enriched by acetate carbon was higher than that in B2 cells (Figure 3A). Furthermore, citrate levels doubled in B1a stimulated with acetate (Figure 3A), suggesting an increased TCA cycle flux.

We hypothesized that acetate would subsequently promote oxidative phosphorylation (OXPHOS). We showed that acetate treatment alone increased the maximal respiratory capacity of cells as measured by oxygen consumption rate (OCR) after addition of FCCP (Figure 3B) (2-way ANOVA, *P* < 0.001 in B1a cells and *P* < 0.05 in B2 cells). We also confirmed that B1a cells were metabolically more active than B2 cells under unstimulated conditions as previously reported (30), demonstrated by increased basal extracellular acidification rate (ECAR; Supplemental Figure 3F), and that acetate treatment significantly increased overall OCR (*P* < 0.0001 for time x group interaction by 2-way ANOVA), which is driven by changes to maximal OCR (Figure 3B).

To determine whether OXPHOS played a role in B10 cell differentiation, we incubated cells with oligomycin, a potent inhibitor of ATP synthase, in the presence or absence of acetate. The proportion of B1a cells expressing IL-10 when incubated with a high dose of oligomycin (1 μM; Figure 3C) as well as a low dose of oligomycin (5 nM; Supplemental Figure 3G) was drastically reduced compared with unstimulated cells, independent of the effect on cell viability (Supplemental Figure 3, H and I). This suggests that energy production from OXPHOS was required for B1a cells to produce IL-10 under basal condition. However, addition of acetate in the presence of oligomycin still promoted IL-10 production, suggesting that other mechanisms were also involved. Because glycolysis and β-oxidation of fatty acids are alternative sources of acetyl-CoA, we used glycolysis and β-oxidation inhibitors to confirm the importance of acetyl-CoA on basal IL-10 production. Etomoxir, which inhibits the conversion of fatty acid in acetyl-CoA, significantly decreased the basal IL-10 production (Figure 3D), confirming the critical role of acetyl-CoA for IL-10 production. We note that although data presented were on live-gated cells, the metabolic inhibitors etomoxir had a slight but significant effect on cell viability, whereas oligomycin significantly affected cell viability (Supplemental Figure 3H). On the other hand, the addition of 2-deoxyglucose (2-DG), a glycolysis inhibitor, had no impact (Figure 3D). Treatment with acetate in the presence of these inhibitors induced a significant increase of IL-10, likely by increasing the acetyl-CoA pool (Figure 3D).

**Figure 4:**
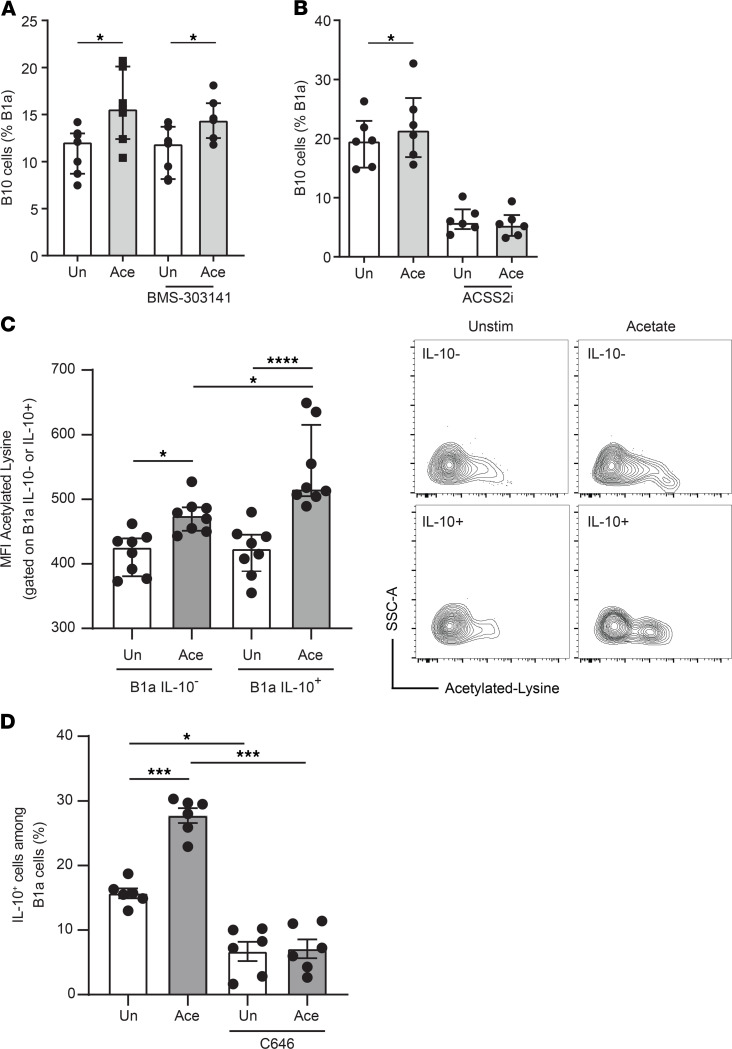
Acetate promotes B10 cells via protein acetylation. 10^6^ peritoneal cells were incubated overnight with 15 μM ACLY inhibitor BMS-303141 (**A**) or 10 μM ACSS2 inhibitor (**B**) in the presence or absence of 10 mM acetate; proportion of B10 cells among B1a cells quantified by flow cytometry (*n* = 6–8). (**C**) MFI of total acetylated lysine was determined from IL-10^–^ and IL-10^+^ B1a cells by flow cytometry in the presence or absence of 10 mM acetate (*n* = 8). (**D**) 10^6^ peritoneal cells were incubated overnight with 18 μM P300 inhibitor C646 in the presence or absence of 10 mM acetate (*n* = 6). Results are represented as median (IQR) with *P < 0.05, ***P < 0.005, and ****P < 0.001 by Mann Whitney *U* or 1 way-ANOVA. Results were confirmed in 2 to 3 independent experiments. ACLY, ATP citrate lyase; Un, unstimulated; MFI, median fluorescence intensity.

The positive effect of acetate on IL-10 production, despite the blockade of mitochondrial metabolism, suggests that other mechanisms of action unrelated to the TCA cycle were also involved. Acetyl-CoA is a substrate for protein acetylation, a posttranslational reversible modification of proteins. Tight control of protein acetylation regulates cell metabolism and signaling by controlling protein interactions, activity, and localization (19). Acetate can be converted into acetyl-CoA either directly by the enzyme ACSS member 2 (ACSS2) or indirectly from mitochondrial-derived citrate by ACLY. The inhibition of ACLY with BMS-303141 affected neither the basal expression of IL-10 by B cells nor the acetate-induced B10 cell differentiation (Figure 4A), suggesting that the increased citrate in B1a cells in the presence of acetate (Figure 3A) was mainly used to fuel the TCA cycle. On the contrary, the inhibition of ACSS2 significantly reduced the proportion of B10 cells under basal condition and abrogated the effects of acetate (Figure 4B), showing that this enzyme was critical for acetate-mediated B10 cell differentiation.

Because ACSS2-produced acetyl-CoA is a substrate for protein acetylation, we next assessed the impact of acetate on protein acetylation by flow cytometry. Acetate treatment significantly increased lysine acetylation in B1a cells compared with controls, but this effect was particularly exacerbated in B1a IL-10 producers (Figure 4C). Protein acetylation is mediated by lysine acetyl transferase (KAT) with the KAT P300 known to contribute to IL-10 production in Tregs (31). To determine the role of P300 in B10 cell differentiation, we stimulated peritoneal cells overnight with C646, a specific inhibitor of P300, and found that like in Tregs, the inhibition of P300 decreased basal production of IL-10 by B cells (Figure 4D). The effects of acetate were abrogated in the presence of P300 inhibitor, showing the key role of this enzyme in acetate-mediated B10 cell differentiation (Figure 4D). Together, these results show that conversion of acetate into acetyl-CoA was used for energy production and cytoplasmic protein acetylation, both necessary for IL-10 production by B cells.

### Acetate promoted B10 cell differentiation from human PBMCs.

**Figure 5:**
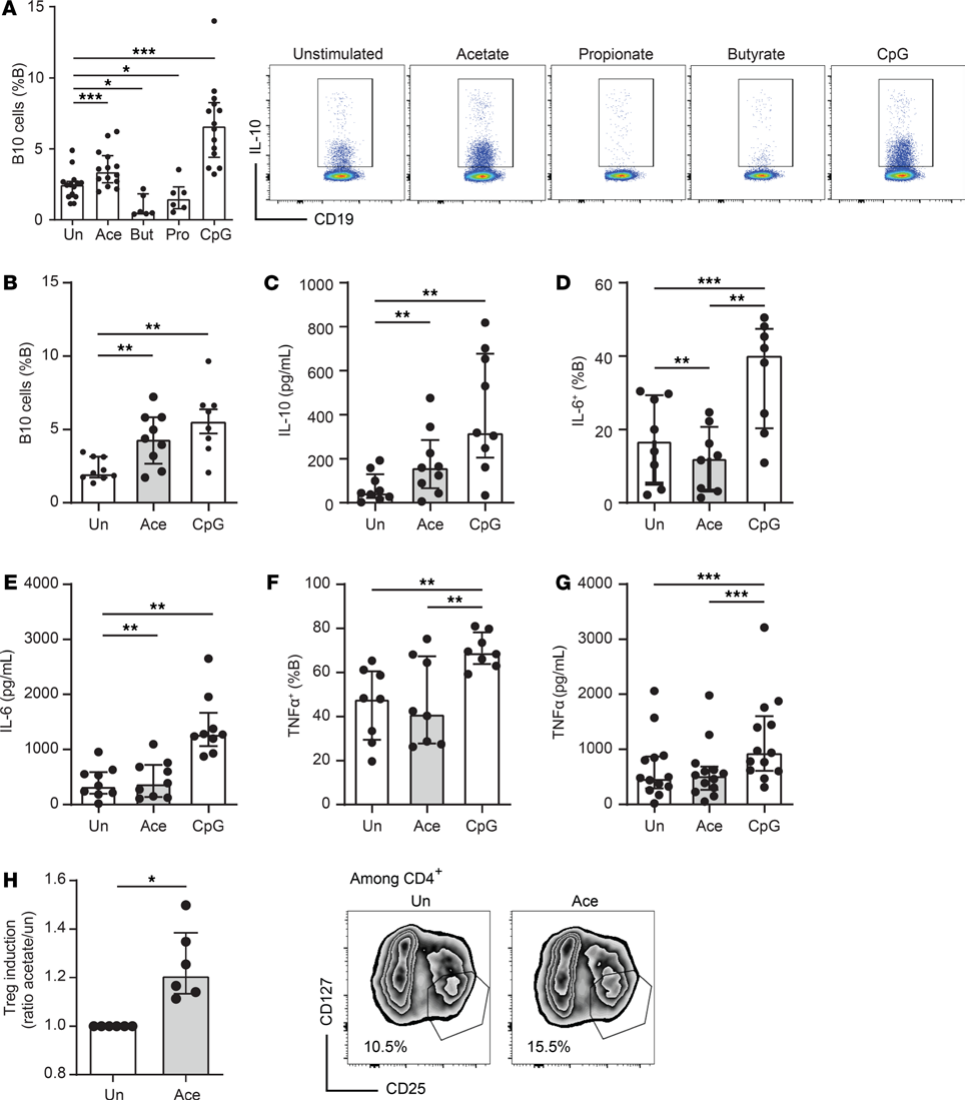
Acetate promotes human B10 cells in peripheral blood. (**A**) Overnight culture of human PBMCs (*n* = 6–14) with acetate (Ace 10 mM), butyrate (But 1 mM), propionate (Pro 1 mM), or 1 μM CpG. B10 cells were assessed among B cells as IL-10^+^CD19^+^ cells by flow cytometry (*n* = 6–14). (**B**) Isolated B cells from PBMCs were incubated overnight with or without 10 mM acetate on CD40 ligand–coated plates, CD19^+^IL-10^+^ B cells assessed by flow cytometry, and (**C**) quantification of IL-10 was performed by ELISA from culture supernatants (*n* = 8–13). (**D–G**) TNF-α^+^CD19^+^IL-6^+^ B cells were assessed by flow cytometry and quantification of IL-6 and TNF-α by ELISA. (**H**) Naive T cells were sorted and cultured for 3 days with plate bound anti-CD3 (dose 1 μg/mL) in the presence of B cells previously incubated overnight with 10 mM acetate or of unstimulated B cells (ratio 1:1) (*n* = 6). Tregs were assessed as CD4^+^CD25^hi^CD127^lo/–^. Wilcoxon paired tests were used with *P < 0.05, **P < 0.01, and ***P < 0.005. All results are represented as median (IQR). Results were confirmed in 2 independent experiments. Un, unstimulated.

We next aimed to determine whether the effects of acetate on B10 cell development in mice were applicable to human B cells. Because the peritoneal cavity is the main source of B1a and B10 cells in mice, we first wanted to assess whether this was also the case in humans. We collected peritoneal cells from subjects who underwent coelioscopy for exploration of ovarian cyst or peritoneal dialysis and found that B cells were poorly represented in the human peritoneal cavity (Supplemental Figure 4A) and nonresponsive to acetate (Supplemental Figure 4B). However, we identified that the treatment of PBMCs with acetate overnight induced B10 cells (Figure 5A; gating strategy presented in Supplemental Figure 4C). Like in mice, this effect was direct because acetate induced B10 cell differentiation from isolated B cells (Figure 5B) and an increased IL-10 production was shown by ELISA (Figure 5C). This effect was mainly observed in CD24^hi^CD27^+^ B cells with only a trend in CD24^hi^CD38^hi^ B cells, but not in CD5^+^ B cells or CD43^+^CD27^+^ B1 equivalent human cells (Supplemental Figure 4D). Thus, contrary to mice, mostly non–B1a CD24^hi^CD38^+^ and CD27^+^ cells produced IL-10 when stimulated with acetate. Interestingly, acetate specifically induced IL-10 with no effect on other cytokines such as IL-6 and TNF (Figure 5, D–G); conversely, CpG increases both IL-10 and proinflammatory cytokines TNF-α and IL-6 as previously shown by us (32). Moreover, B10 cells play a central role in immune tolerance by inducing Tregs. To determine whether acetate promoted functional B10 cells with such promoting effects, we cocultured B cells preincubated with acetate or PBS with naive T cells. We found that differentiation toward Tregs was significantly increased (Figure 5H). These results show that the effect of acetate on B10 cell differentiation was not limited to mice but was also applicable in humans.

### Acetate promoted human B10 cells through metabolic changes and protein acetylation.

**Figure 6:**
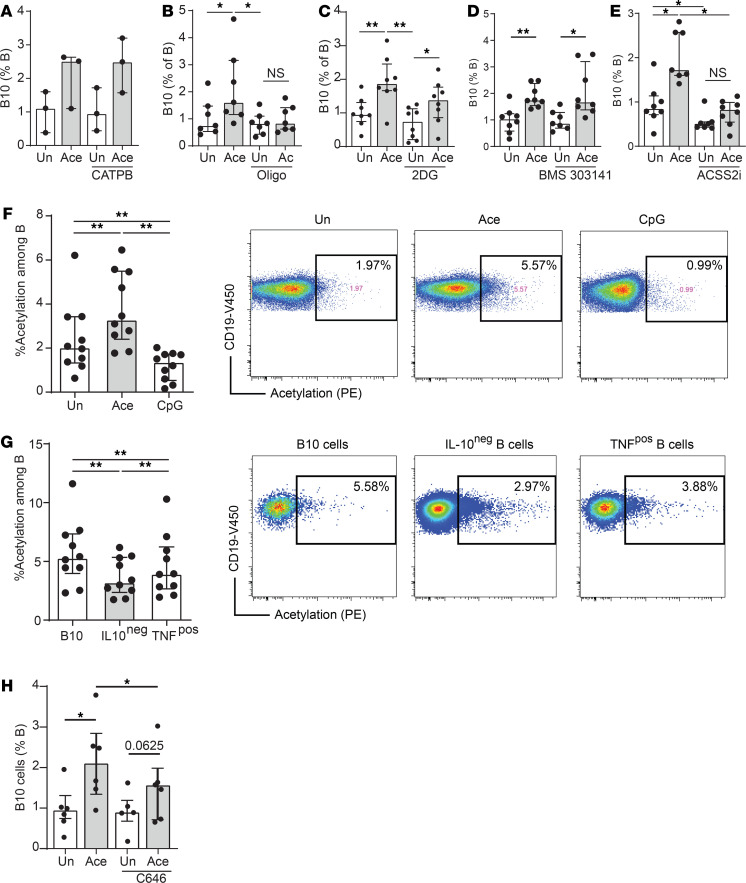
Human B cells share similar metabolic requirements with mouse B cells for B10 cell induction. (**A**) 10^6^/mL of PBMC-sorted B cells were cultured overnight with 10 mM acetate in the presence or absence of 10 μM of CATPB (GPR43 antagonist) and the proportion of B10 cells was characterized by flow cytometry (*n* = 3). (**B–D**) To determine whether the induction of human B10 cells by acetate was also dependent on the TCA cycle, glycolysis and ACSS2-dependent pathways (**B**) oligomycin (1 μM), (**C**) 2-DG (5 mM), (**D**) ACLY inhibitor BMS 303141 (15 μM), and (**E**) ACSS2i (20 μM) were added to the culture in the presence or absence of acetate (10 mM) overnight, and the proportion of B10 cells was characterized by flow cytometry (*n* = 7–8). (**F** and **G**) Lysine-acetylation was assessed by flow cytometry using anti-acetyl lysine antibody (7F8) (*n* = 10). (**H**) C646, a P300 inhibitor, was added at 30 μM in the presence or absence of acetate (10 mM) overnight, and the proportion of B10 cells was characterized by flow cytometry (*n* = 6). Results are represented as median (IQR) with *P < 0.05 and **P < 0.01 by Wilcoxon paired tests. Results were confirmed in 2 independent experiments. ACSS2, acetyl-CoA synthetase 2; ACSS2i, ACSS2 inhibitor; ACLY, ATP citrate lyase; Un, unstimulated.

To determine whether the effect of acetate on human cells involved similar mechanisms as those observed in mice, we focused on the impact of acetate on cell metabolism and protein acetylation. Of note, as in mice, acetate did not mediate its effects via GPR43 because acetate treatment in the presence of CATPB, a specific inhibitor of GPR43, did not affect the positive effect of acetate on B10 cell differentiation (Figure 6A). On the other hand, as observed in mice, human B cells treated with acetate in the presence of oligomycin (Figure 6B) as well as in the presence of 2-DG (Figure 6C) significantly decreased the effect of acetate, suggesting that metabolic activation was necessary. As observed in mice, the inhibition of ACLY, an enzyme that converts acetyl-CoA from the TCA cycle, did not affect the induction of B10 cell by acetate, whereas the blockade of ACSS2, which converts acetate into acetyl-CoA, decreased acetate-induced B10 cells (Figure 6, D and E). This conversion of acetate into acetyl-CoA was also associated with an increase of protein lysine acetylation as shown by flow cytometry under acetate treatment conditions (Figure 6F), suggesting that the conversion of acetate into acetyl-CoA could be used for the acetylation of cytoplasmic protein. In addition, B10 cells had significantly more lysine-acetylated proteins than IL-10^neg^ B cells or TNF^+^ B cells (Figure 6G). Interestingly, CpG did not show such effects, suggesting a different mechanism of action. As in mice, human P300 is a cytosolic enzyme involved in protein acetylation. Its inhibition in the presence of acetate significantly decreased the effects of acetate on B10 cell induction (Figure 6H), showing that this enzyme and thus protein acetylation contributed to the effects of acetate. We investigated the impact of acetate on acetylation of protein known to promote IL-10 once acetylated in other immune cell subsets, which are β-tubulin (33) and glycogen-sensing kinase 3 (GSK3) (34, 35). We found that acetate induced tubulin acetylation in both mouse and human B cells IL-10 producers and nonproducers by flow cytometry, suggesting that, in our model, acetate-induced β-tubulin acetylation was not specifically linked to IL-10 (data not shown). We also found that acetate did not induce GSK3 acetylation, assessed by Western blot, on sorted human and mouse B cells, suggesting that this pathway was not involved (data not shown). These results suggest that the potential protein targeted might have been a target protein involved in IL-10 production. Further investigations would be required to identify this target.

Together these results show that, as in mice, acetate promoted human B10 cell differentiation through energy metabolism and protein acetylation by the enzyme ACSS2.

### Increase of acetate in vivo was correlated with increased B10 cells in healthy participants in vivo.

**Figure 7:**
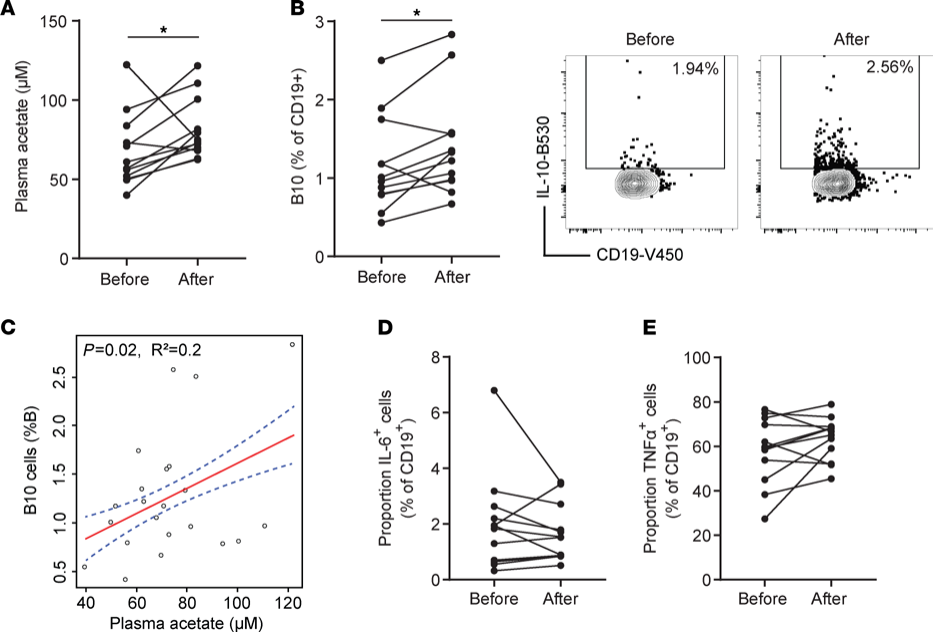
One week of dietary fiber supplementation increases plasma acetate and B10 cells in healthy participants. (**A**) A 4-hour fasting plasma concentration of acetate was determined by ^1^H-NMR spectroscopy at baseline and after 7 days of FibreMax supplementation. (**B**) The proportion of B10 cells among CD19 cells was determined by flow cytometry at baseline and after 7 days of FibreMax supplementation. (**C**) The correlation between plasma acetate concentration and proportion of B10 cells among CD19^+^ PBMC cells before and after supplementation was determined by linear regression. The proportion of cells expressing (**D**) IL-6 and (**E**) TNF among CD19 cells was determined by flow cytometry at baseline and after 7 days of FibreMax supplementation. Results are represented as median (IQR) with *P < 0.05 by Wilcoxon paired tests from *n* = 12 participants.

To determine the effect of acetate on B10 cells in humans in vivo, we used a short-term dietary fiber intervention, a safe and effective method to increase acetate. Soluble dietary fibers are fermented by gut bacteria into SCFAs, with acetate levels being raised in the peripheral blood (13), where we identified human B10 cells. To determine whether dietary fibers could promote B10 cell differentiation, we enrolled *n* = 12 healthy woman volunteers to consume 33.75 g of FibreMax (equivalent to 18.7 g of soluble fiber) daily for 7 consecutive days. Plasma acetate, quantified by NMR, was significantly elevated 7 days after dietary fiber supplementation (Figure 7A), confirming the efficacy of the intervention. To determine whether the proportion of B10 cells was impacted by this intervention, we stimulated PBMCs isolated from participant preintervention and postintervention with ionomycin and phorbol 12-myristate 13-acetate (PMA) in vitro for 4 hours. One week of dietary fiber supplementation was sufficient to significantly increase the proportion of B10 cells as shown by flow cytometry (Figure 7B). This finding suggests that the rise of acetate in vivo might have promoted the induction of B10 cells in the blood, which is supported by the positive correlation between plasma acetate concentration and proportion of B10 cells (*P* = 0.02, R^2^ = 0.2; Figure 7C). On the other hand, neither IL-6– (Figure 7D) nor TNF-α–producing (Figure 7E) B cells were affected by this dietary fiber intervention. These data show that dietary fiber intervention in healthy participants increased plasma acetate and promoted B10 cell differentiation.

## Discussion

Although the induction of B10 cells by microbe-associated molecular pattern (particularly CpG) is well characterized, we show here that acetate also directly promoted mouse and human B10 cells both in vitro and in vivo. We identified that the mechanisms were conserved between the 2 species and that the conversion of acetate into acetyl-CoA mediated B10 cell differentiation through 2 complementary mechanisms: (a) the increase of energy availability by fueling the TCA cycle and OXPHOS and (b) protein acetylation via ACSS2. Acetate-induced B10 cells were functional, by alleviating arthritis when adoptively transferred into mice and by inducing Treg differentiation of naive T cells in humans. We identified that short-term dietary fiber supplementation in humans increased plasma acetate as well as circulating B10 cells, offering a safe and effective intervention to potentially restore B10 cells in autoimmune diseases with B10 cell defects such as rheumatoid arthritis.

The host–microbiota interaction is complex and a full understanding on how the host tolerates the gut microbiota is slowly coming to light. Among the mechanisms of tolerance, the induction of Tregs by SCFAs has been reported (14–16). In the present work, we report that acetate promoted B10 cells from B1a cells in mice. Contrary to its effect on Treg induction, we found that acetate did not promote B10 cells via GPCR stimulation or epigenetic changes in the IL-10 promoter. This highlights that gut bacterial metabolites promoted tolerance through multiple mechanisms.

A surprising result was that butyrate had a direct inhibitory effect on B10 cell development, whereas it is a potent inducer of Tregs in the colon (22). This effect could account for the beneficial effect of butyrate in colorectal cancer because the presence of B10 cells in solid tumors has been linked to a decrease in antitumor immune response (36). Butyrate is mostly localized in the colon, which may be one mechanism by which site-specific regulation of specific immune subsets like B10 cell occurs. Indeed, a previous report on the induction of splenic B10 cell by butyrate was shown to be an indirect effect by promoting the serotonin-derived metabolite 5-hydroxyindole-3-acetic acid (20). In our hands, propionate had no direct effect on B10 cell induction, whereas it has been shown that LPS- or CpG-stimulated splenic B cells could promote IL-10 production in response to propionate (21). This highlights that different SCFAs elicit distinct and specific effects on B10 cell development depending on the environmental context. Our finding that acetate directly induced B10 cell without the need for prior TLR activation has therapeutic potential without the risk of eliciting an uncontrolled inflammation. Indeed, under basal conditions, acetate is typically the only SCFA detectable in the periphery (outside the colon and liver) at concentrations high enough to have physiological effect on cells (13).

These differential effects of SCFAs on B10 cell differentiation were conserved from mouse to humans with acetate alone having promoting effects through similar mechanisms. In mice, only B1a but not B2 cells differentiated into B10 cells under acetate stimulatory conditions. This is even more surprising given the relative incorporation of carbon, derived from acetate, into TCA cycle intermediates was the same in both cell subsets. The difference of effects of acetate might lay in the fact that B2 cells have a substantially lower metabolic activity than B1a cells, which suggests that a threshold of energy might be necessary for B cells to differentiate into B10 cells. This higher metabolic activity has previously been shown to be associated with a higher number of mitochondria in B1 cells (30), which could explain the predominant effect of acetate on this subset. Acetate induced B10 cell differentiation from non–B1 B cells in humans, particularly CD24^hi^CD27^+^ B cells, through similar mechanisms, involving metabolic activation and protein acetylation. Similar to B1a cells in mice, these cells might reach a specific energetic threshold in the presence of acetate necessary for IL-10 production. Our result suggests that both metabolic activation and protein acetylation may be a prerequisite for B10 cell induction. To our knowledge, this is the first evidence that ACSS2 contributes to B10 cell development. These results also suggest that acetate-induced B10 cells might be an evolutionary preserved strategy to maintain tolerance toward commensal bacteria in both mice and humans.

The availability of acetyl-CoA regulates posttranslational lysine acetylation in both histone and nonhistone proteins. This posttranslational modification is a key regulator of protein function in many biological processes either inhibiting or activating enzyme activity (19). We showed that the inhibition of the KAT p300 decreased acetate-induced B10 cells, confirming the importance of acetylation in acetate-induced B10 cells. The activity of several cytoplasmic proteins is regulated by acetylation and has been reported to promote IL-10 production. Among these candidates, stabilization of β-tubulin by acetylation has been shown to promote IL-10 production in macrophages (33), and the inhibition of the metabolic checkpoint GSK3 by acetylation (35, 37) promotes IL-10 production in T cells and dendritic cells (34, 38). Although these proteins were not targeted by acetate, further investigations are required to identify which candidates are acetylated by acetate treatment, leading to B10 cell development.

We have also established that acetate promoted B10 cells in vivo in both humans and mice. Because the B10 cell precursors are located in different sites in these 2 species, we used different strategies to increase acetate in proximity to B10 cell precursors. We found that the administration of acetate both acutely through i.p. injections and chronically in drinking water were effective methods to promote B10 cells in mice. In our human study, we used a mixture of different dietary fibers to increase acetate in blood. It would thus be necessary to determine which particular types of fibers are optimal for promoting B10 cells. This finding is clinically relevant because dietary intervention to increase acetate or direct supplementation with acetate (39), which are shown to increase circulating acetate, could be safe and cost-effective interventions to restore B10 cells in diseases such as multiple sclerosis (40) or rheumatoid arthritis (7).

Our work highlights a mechanism to promote B10 cell development. Such knowledge may allow safe and cost-effective therapeutics involving the use of acetate or dietary intervention to tackle diseases in which B10 cells are defective. This pathway we identify here, a product of dietary fiber (acetate) promoting antiinflammatory B10 cells, may explain the rise in inflammatory diseases in fiber-underconsuming Western populations.

## Methods

### Animals.

All mice were on a C57BL/6 background and maintained under specific pathogen–free conditions in the mouse facility of the Charles Perkins Center. Experiments on WT mice were performed on 5- to 8-week-old male C57BL/6J mice that were obtained commercially (Animal Bioresources). *Gpr43*^–/–^ mice (Deltagen) were crossed to a C57BL/6 background greater than 13 generations.

### In vitro study of mouse cells.

Peritoneal lavage was performed using 5 mL of cold PBS with 2% FBS (Thermo Fisher Scientific, Australian origin) to obtain peritoneal cavity B cells. Splenocytes were isolated after mechanical dissociation of spleen and treatment with RBC lysis buffer according to the manufacturer’s instructions (BioLegend). Peritoneal and splenic cells were resuspended in complete media composed of RPMI 1640, 1× Penicillin/Streptomycin/Glutamine, 1 mM Sodium Pyruvate, 10 mM HEPES (all from Gibco), 10% FBS (Thermo Fisher Scientific), and 55 μM of β-mercaptoethanol and seeded at 10^7^ cells/mL into each well. Cells were cultured overnight in the presence or absence of acetate (10 mM), butyrate (1 mM), and propionate (1 mM) (MilliporeSigma) and for the last 4 hours with PMA (50 ng/mL, MilliporeSigma), ionomycin (500 ng/mL, MilliporeSigma), and Golgi-Plug (BD Biosciences). Fc receptors were blocked with TruStain fcX (anti-mouse CD16/32 antibody; BioLegend). Cells were permeabilized with Cytofix/Cytoperm buffer (BD Biosciences) and 1× Perm/wash buffer (BD Biosciences) according to the manufacturer’s instructions and stained with BV785-conjugated anti-CD45 (30-F11), FITC-conjugated anti-CD19 (6D5), PE/Cy7-conjugated anti-CD5 (53-7.3), APC-conjugated anti-CD23 (B3B4), PE-conjugated anti–IL-10 (JES5-16E3) antibody or PE-conjugated isotype control (RTK4530), or PE-conjugated acetylated-lysine (15G10), APC-conjugated anti–IL-10 (JES5-16E3) antibody, or APC-conjugated isotype control (RTK2758), all purchased from BioLegend. For Treg identification, PerCP/Cy5.5-conjugated anti-CD4 (GK1.5), BV605-conjugated anti-CD25 (PC61; BioLegend), and APC-conjugated anti-FoxP3 (FJK-16s; eBioscience) were used. Compensations were performed using Ultra-Comp eBeads (eBioscience). Flow cytometry was done with the LSRII (BD Biosciences) at the Sydney cytometry facility and analysis with the software FlowJo V10 (Treestar). Cells were gated based on forward scatter (FSC) and side scatter (SSC), followed by exclusion of doublets and dead cells (LIVE/DEAD positive using Fixable Blue Dead Cell Stain Kit, eBioscience).

### Diets and SCFA treatments.

AIN93G diets were purchased from Specialty Feeds. Sodium acetate, propionate, or butyrate (MilliporeSigma) were administered in drinking water at 200 mM, 100 mM, and 100 mM, respectively, for 3 weeks before the experiment. PBS or pH-adjusted sodium acetate (500 mg/kg) were administered i.p. at 6 pm and 6 am the next day and animals were culled 4 hours later.

### Quantification of SCFA concentrations from cecal contents by NMR.

Briefly, cecal contents were homogenized and filtered through a 3kDa membrane and metabolites extracted from the aqueous phase of water/chloroform/methanol mixture. Samples are analyzed on a Bruker 600MHz NMR containing 4,4-dimethyl-4-silapentane-1-sulfonic acid as internal standard.

### Biodistribution of acetate.

Mice were fasted 6 hours before the PET scan. Mice, anesthetized with 4% (v/v) isoflurane and maintained at 1%–2%, were scanned using a small-animal Inveon PET/CT scanner (Siemens) following previously described methods (41–43). Body temperature was maintained with a feedback-regulated heating pad and respiration was monitored (BioVet; m2m Imaging Corp.). Scans started with the administration of ^11^C-acetate (50–100 MBq) i.v. into the tail vein or colonically by using tubing placed 1 cm into the rectum, and after 90 minutes of PET imaging, data acquisition concluded with a 10-minute CT scan for anatomical coregistration information. ^11^C-acetate was produced as previously described (44).

Image reconstruction was performed using IAW 2.02 (Siemens). The list-mode data was histogrammed into 18 frames (6 × 10 s, 4 × 60 s, 1 × 300 s, 5 × 600 s, 2 × 900 s) for the period 0–90 minutes after tracer injection. Emission sinograms were reconstructed using a two-dimensional filter back projection with a zoom of 1.5. The reconstructed images consisted of 18 frames of a 128 × 128 × 159 matrix with a voxel size of 0.52 × 0.52 × 0.796 mm^3^. They were corrected for attenuation (CT-based), scatter, randoms, normalization, isotope decay, branching ratio, and deadtime and were calibrated to Bq/mL. Reconstructed PET images were then registered onto the CT images. PET signals on the desired region of interest were obtained over the scan period (1.5 hours) using IAW 2.02 (Siemens).

For biodistribution studies, animals were sacrificed 30 minutes or 90 minutes after the administration of ^11^C-acetate (6–12 MBq) i.v. into the tail vein or colonically by using tubing placed 1 cm into the rectum. Organs of interest were harvested, weighted, and counted in the γ counter (2480 Wizard2, PerkinElmer). The percentage of injected dose per gram of tissue (%ID/g) was determined by decay correction of the radiotracer for each sample normalized to a standard of known weight, which was representative of the injected dose.

### Arthritis model and B cell transfer.

Arthritis was induced on day 0 using 4 mg (200 μl) of ArthritoMab Monoclonal Antibody Cocktail for C57BL/6 administered i.p. (CIA-MAB-2C, MD Biosciences). Two hours later, 200 μl of 10^6^ acetate-stimulated B cells or 10^6^ nonstimulated B cells resuspended in PBS plus 2% FBS or 200 μl of PBS plus 2% FBS were injected i.p. On day 3, 50 μg of LPS (0.5 mg/mL) were administered i.p. Joints were blindly monitored daily by 2 investigators and mice were culled on day 7. To assess the effect of acetate, acetate was administered in drinking water at 200 mM, 3 weeks prior and throughout the experiment. For B10 cell adoptive transfer experiments, on day 2, peritoneal cells were cultured overnight in the presence or absence of acetate (10 mM). On day 1, B cells from nonstimulated and acetate-stimulated peritoneal cells were sorted using Pan B Cell Isolation Kit II (Miltenyi Biotec) according to the manufacturer’s instructions.

### Mass spectrometry and ^13^C-acetate tracing.

A solution of 1 × 10^6^ B1a or B2 cells was incubated with or without ^13^C-acetate (10 mM; MilliporeSigma) for 6 hours. Immediately afterwards, cells were washed twice with ice-cold phosphate-free saline (0.9% w/v sodium chloride) and cell pellet frozen in a dry ice–methanol bath and stored at –80^o^C until ready for analysis. Cellular metabolites were extracted using 100 μl of cold (–30°C) extraction buffer consisting of 2:2:1 (volume) mixture of acetonitrile, methanol, and water, and of internal standards deuterated thymine and D-camphor-10-sulfonic acid. Cell slurry was first incubated on ice bath for 10 minutes with intermittent vortexing and then centrifuged for 10 minutes at 15,000*g* and 4°C. Supernatant was transferred into fresh tube and dried to completeness without heat by speed-vac. Dried metabolites were resuspended in 20 μL H2O and then transferred into HPLC vials.

Two LC-MS analyses were performed to measure central carbon metabolites using an Agilent Infinity 1260 LC coupled to an AB Sciex QTRAP5500 MS. The “reverse-phase” method consisted of injecting a 5-μL sample into a Synergi 2.5 μm 100 Å Hydro-RP column (2.0 mM I.D., 100 mM length; Phenomenax), with mobile phase comprised of buffers 97:3 (v/v) water/acetonitrile containing 10 mM tributylamine and 15 mM acetic acid, and 100% acetonitrile (45). The “amide” method consisted of injecting a 2.5-μL sample into an XBridge Amide 3.5 μm column (2.1 mM I.D., 100 mM length; Waters), with mobile phase comprised of buffers 95:5 (v/v) water/acetonitrile containing 20 mM ammonium hydroxide and 20 mM ammonium acetate, and 100% acetonitrile (46). Mobile phase flow rate was 250 μL/minute. Ion source temperature and spray voltage were set at 350°C and 4500V, respectively, for both positive and negative modes. Multiple reaction monitoring acquisition was performed to measure the abundance of metabolites, and the 13C enrichments of some nonessential amino acids, glycolytic, and TCA metabolites. For data extraction and analysis, raw instrument data files were converted into text files using MSConvert before being analyzed using in-house MATLAB scripts (47).

### Cellular bioenergetic assay.

Both OCR and ECAR were measured on the Seahorse XFe96 analyzer. B1a or B2 cells were sorted and plated at a density of 0.5 × 10^5^ in bicarbonate-free DMEM (MilliporeSigma) supplemented with 10 mM glucose, 1 mM sodium pyruvate, 2 mM L-glutamine, and 5 mM HEPES. Culture plate was coated with 100 μg/mL Poly-D-Lysine (MilliporeSigma) to assist adherence of cells. Mitochondria functions were assessed by sequential injection of Oligomycin (2 μM), FCCP (1 μM), and Rotenone plus Antimycin A (1 μM each) (all from MilliporeSigma). In some experiments, purified B1a or B2 cells were treated with or without acetate (10 mM) overnight before measurement of OCR/ECAR. In each experiment, 5 replicates were used for each group.

### Assessment of metabolic pathways on mouse B10 cell development.

To assess the metabolic pathways required for B10 cell development, 1 × 10^6^ total IP cells were incubated with or without acetate (10 mM) in the absence of CpG along with BMS-303141 (15 μM; MilliporeSigma), Acetyl-CoA Synthetase 2 Inhibitor (10 μM; Calbiochem), oligomycin (1 μM; MilliporeSigma), Etomoxir (200 μM; MilliporeSigma), or 2-DG (1 mM; MilliporeSigma). For assessment of the role of acetylation in promoting B10 cell development, C646 (18 μM; MilliporeSigma) was used. B cells were analyzed after overnight incubation for the production of IL-10.

### Human study.

Blood was collected in EDTA tubes. PBMCs were isolated from whole blood collected in EDTA tubes using Ficoll-Paque Plus (GE Healthcare). Peritoneal cells of patients who underwent coelioscopy for exploration of ovarian cyst or peritoneal dialysis were harvested by peritoneal lavage. Peritoneal cells were collected by centrifugation and RBC lysis performed using ACK lysis buffer (Miltenyi Biotec). B cells are isolated using Rosette Sep Human B cell enrichment (Stemcell Technologies) followed by Ficoll separation from fresh blood. Cells were cultured in RPMI 1640 (Life technologies) with 10% FBS with Penicillin (100 UI/mL, MilliporeSigma) and Streptomycin (100 UI/mL, MilliporeSigma) for 24 or 72 hours on 96-well plates at 1.5 × 10^6^ cells/mL and 200 μl per well. PBMCs were activated overnight with CpG (TLR9 ligand, ODN 2006; InvivoGen) acetate (10 mM), butyrate (1 mM), or propionate (4 mM) in the presence or absence of 10 mg/mL CpG. In the case of B cells isolated using RosetteSep Human B Cell Enrichment Cocktail (Stemcell Technologies), stimulations were done on CD40L-coated well (1 mg/mL). Naive T cells, defined as CD4^+^CD25^–^ T cells, were isolated by flow cytometry (FACSaria). They were cultured with plate bound anti-CD3 (dose 1 μg/mL) during 72 hours with autologous B cells (in a ratio 1:1) either previously stimulated for 24 hours with acetate or unstimulated. For Treg staining, FITC-conjugated anti-CD4 (RPA-T4, BD Pharmingen), PE-conjugated anti-CD25 (BC96, eBioscience), and PE-Cy5-conjugated anti-CD127 (eBioRDR5, eBioscience) antibodies were used. Dead cells were excluded using DAPI staining. Tregs were defined as CD4^+^CD25^+^CD127^lo/–^.

CATBP (Tocris), a human GPR43 antagonist, was resuspended at 50 mM in DMSO and used at a final concentration of 10 μM. 2-DG was used at 5 mM. CATPB (1 μM; Tocris), oligomycin (1 μM; MilliporeSigma), BMS-303141 (15 μM; Tocris), ACSS2i (20 μM; Selleckchem), and C646 (30 μM; MilliporeSigma) were added to the culture in the presence or absence of acetate (10 mM).

PMA (0.1 μg/mL; BioVision) and ionomycin (0.5 μg/mL; BioVision) were added for the last 4 hours and brefeldin A (10 μg/mL) for the last 2 hours. B cells were stained with V450-conjugated anti-CD19 (HIB19, BD Pharmingen) and Fixable Viability Dyes eFluor506 (eBioscience), permeabilized with Cytofix/Cytoperm buffer (BD Biosciences) and 1X Perm/Wash buffer (BD Biosciences) according to the manufacturer’s instructions, and intracellularly stained with APC-conjugated anti–IL-10 antibody (JES3-19F1) or APC-conjugated isotype control (R35-95, BD Pharmingen).

Lysine-acetylation was assessed using PE-conjugated anti-acetyl lysine antibody (7F8, Abcam) at 3 μg/mL in 1X Perm/Wash buffer cells. Flow cytometry was performed on FACSCanto II (BD Bioscience) and analyzed with FlowJo software 6.3 (Treestar). PBMCs were gated based on FSC and SSC, followed by exclusion of doublets and dead cells. B cells were gated according to CD19-positive expression. B10 cells were gated according to IL-10-positive expression.

### Dietary fiber intervention.

The dietary fiber supplement FibreMax (New Image) contains fiber from the following sources: chicory root extract (47%), psyllium husk (23.5%), soy fiber (23.5%), oat bran (5%), and pectin (1%). Each participant was provided with a sealed tin containing 420 g of the product and a measuring scoop. Participants were instructed to consume 3 servings (total 33.75 g/day) of the supplement for 7 days. Compliance was ascertained by weighing leftover at final assessment clinic. For B10 cell analysis, PBMC was isolated from collected blood using the SepMate-50 and Lymphoprep density gradient medium (Stemcell Technologies) and stored in cryopreservative medium (10% DMSO in FBS). Samples were analyzed in batches using the following antibodies: V450-conjugated anti-CD19 (HIB19), PE-conjugated anti-TNF (Mab11, BD Biosciences) and Vio505-conjugated IL-10 (JES3-9D7), and APC-conjugated anti–IL-6 (REA1037, Miltenyi Biotec). For quantification of SCFAs by NMR, postbreakfast fasting (4 hours) blood samples were collected in 10 mL BD Vacutainer Plastic K2EDTA tubes (Becton Dickinson), centrifuged immediately (3800*g*, 15 minutes at 4°C), and plasma was collected.

### Statistics.

In all figures, medians (IQR) were represented unless otherwise stated in the figure legends. For paired comparisons, we used either Friedman repeated-measures ANOVA for multiple comparisons or Wilcoxon’s tests, depending on the number of conditions studied and whether or not a similar number of samples were available in the different conditions. For unpaired comparisons, we used either Mann-Whitney *U* tests if 2 conditions were available or 1-way ANOVA with Bonferroni’s posttest for multiple comparisons. Two-way ANOVA was performed with Bonferroni’s tests for multiple comparisons for comparison of joint count over time in CAIA mice transferred with B cells. A *P* value of less than 0.05 was considered significant.

### Study approval.

All animal experiments were performed in accordance and with the approval of Sydney Animal Ethic Committee (2014/725 and 2014/667). For the human clinical study, the trial was conducted in accordance with the Declaration of Helsinki, and all procedures were reviewed and approved by the ethics review committee (Royal Prince Alfred Hospital Zone of the Sydney Local Health District (X17-0130 and HREC/17/RPAH/192). The trial was registered with the Australian and New Zealand Clinical Trials Registry (ACTRN12617001139369) and started on the 01/09/2017. All subjects provided written informed consent to participate in the study as approved by the medical ethics committee of Est-I, Dijon, France (no. 2017-A00870-53).

## Author contributions

CID and JT designed the research study, conducted the experiments, acquired and analyzed the data, and wrote the manuscript. RA conducted the experiments, acquired and analyzed the data, and reviewed the manuscript. JM conducted the experiments, acquired and analyzed the data, and reviewed the manuscript. LEQ and JRK participated to the experiments and reviewed the manuscript. AA participated in the experiments and reviewed the manuscript. MD provided peritoneal cells from patients and reviewed the manuscript. GP participated in the experiments and reviewed the manuscript. DN helped with data analyses. MCBA recruited participants for the human fiber study. FS, AP, GP, RR, MJA, and SH participated in the experiments. AKG helped design and registered the human fiber study, recruited and coordinated the participants for dietary intervention, and reviewed the manuscript. SJS gave intellectual input and reviewed the manuscript. CRM reviewed the manuscript. RVR conducted human intervention and reviewed the manuscript. LM designed the research study, received funding for the project, conducted the experiments, and wrote the manuscript. CID was designated as the first co–first author because she initiated the project.

## Supplementary Material

Supplemental data

## Version 1 03/17/2021

In-Press Preview

## Version 2 04/08/2021

Electronic publication

## References

[1] Noack M, Miossec P (2014). Th17 and regulatory T cell balance in autoimmune and inflammatory diseases. Autoimmun Rev.

[2] Rosser EC, Mauri C (2015). Regulatory B cells: origin, phenotype, and function. Immunity.

[3] Carter NA (2011). Mice lacking endogenous IL-10-producing regulatory B cells develop exacerbated disease and present with an increased frequency of Th1/Th17 but a decrease in regulatory T cells. J Immunol.

[4] Fillatreau S (2002). B cells regulate autoimmunity by provision of IL-10. Nat Immunol.

[5] Mizoguchi A (2002). Chronic intestinal inflammatory condition generates IL-10-producing regulatory B cell subset characterized by CD1d upregulation. Immunity.

[6] Mauri C (2003). Prevention of arthritis by interleukin 10-producing B cells. J Exp Med.

[7] Daien CI (2014). Regulatory B10 cells are decreased in patients with rheumatoid arthritis and are inversely correlated with disease activity. Arthritis Rheumatol.

[8] Tedder TF (2015). B10 cells: a functionally defined regulatory B cell subset. J Immunol.

[9] Hasan MM (2019). CD24^hi^CD38^hi^ and CD24^hi^CD27^+^ human regulatory B cells display common and distinct functional characteristics. J Immunol.

[10] Barr TA (2007). TLR-mediated stimulation of APC: distinct cytokine responses of B cells and dendritic cells. Eur J Immunol.

[11] Rosser EC (2014). Regulatory B cells are induced by gut microbiota-driven interleukin-1β and interleukin-6 production. Nat Med.

[12] Maslowski KM (2009). Regulation of inflammatory responses by gut microbiota and chemoattractant receptor GPR43. Nature.

[13] Tan J (2014). The role of short-chain fatty acids in health and disease. Adv Immunol.

[14] Arpaia N (2013). Metabolites produced by commensal bacteria promote peripheral regulatory T-cell generation. Nature.

[15] Thorburn AN (2015). Evidence that asthma is a developmental origin disease influenced by maternal diet and bacterial metabolites. Nat Commun.

[16] Tan J (2016). Dietary fiber and bacterial SCFA enhance oral tolerance and protect against food allergy through diverse cellular pathways. Cell Rep.

[17] Kim M (2016). Gut microbial metabolites fuel host antibody responses. Cell Host Microbe.

[18] Balmer ML (2016). Memory CD8(+) T cells require increased concentrations of acetate induced by stress for optimal function. Immunity.

[19] Choudhary C (2014). The growing landscape of lysine acetylation links metabolism and cell signalling. Nat Rev Mol Cell Biol.

[20] Rosser EC (2020). Microbiota-derived metabolites suppress arthritis by amplifying aryl-hydrocarbon receptor activation in regulatory B cells. Cell Metab.

[21] Luu M (2019). The short-chain fatty acid pentanoate suppresses autoimmunity by modulating the metabolic-epigenetic crosstalk in lymphocytes. Nat Commun.

[22] Smith PM (2013). The microbial metabolites, short-chain fatty acids, regulate colonic Treg cell homeostasis. Science.

[23] Bhaskaran N (2018). Role of short chain fatty acids in controlling tregs and immunopathology during mucosal infection. Front Microbiol.

[24] Macia L (2015). Metabolite-sensing receptors GPR43 and GPR109A facilitate dietary fibre-induced gut homeostasis through regulation of the inflammasome. Nat Commun.

[25] Corr M (2011). Interleukin 1 receptor antagonist mediates the beneficial effects of systemic interferon beta in mice: implications for rheumatoid arthritis. Ann Rheum Dis.

[26] Bilotta AJ, Cong Y (2019). Gut microbiota metabolite regulation of host defenses at mucosal surfaces: implication in precision medicine. Precis Clin Med.

[27] Sanchez HN (2020). B cell-intrinsic epigenetic modulation of antibody responses by dietary fiber-derived short-chain fatty acids. Nat Commun.

[28] Graf R (2019). BCR-dependent lineage plasticity in mature B cells. Science.

[29] Kim MH (2013). Short-chain fatty acids activate GPR41 and GPR43 on intestinal epithelial cells to promote inflammatory responses in mice. Gastroenterology.

[30] Clarke AJ (2018). B1a B cells require autophagy for metabolic homeostasis and self-renewal. J Exp Med.

[31] Castillo J (2019). CBP/p300 drives the differentiation of regulatory T cells through transcriptional and non-transcriptional mechanisms. Cancer Res.

[32] Mielle J (2018). IL-10 Producing B cells ability to induce regulatory T cells is maintained in rheumatoid arthritis. Front Immunol.

[33] Wang B (2014). Microtubule acetylation amplifies p38 kinase signaling and anti-inflammatory IL-10 production. Nat Commun.

[34] Hill EV (2015). Glycogen synthase kinase-3 controls IL-10 expression in CD4(+) effector T-cell subsets through epigenetic modification of the IL-10 promoter. Eur J Immunol.

[35] Sarikhani M (2018). SIRT2 deacetylase regulates the activity of GSK3 isoforms independent of inhibitory phosphorylation. Elife.

[36] Shimabukuro-Vornhagen A (2014). Characterization of tumor-associated B-cell subsets in patients with colorectal cancer. Oncotarget.

[37] Jellusova J (2017). Gsk3 is a metabolic checkpoint regulator in B cells. Nat Immunol.

[38] Wang H (2011). The role of glycogen synthase kinase 3 in regulating IFN-β-mediated IL-10 production. J Immunol.

[39] Sugiyama S (2010). Bioavailability of acetate from two vinegar supplements: capsule and drink. J Nutr Sci Vitaminol (Tokyo).

[40] Knippenberg S (2011). Reduction in IL-10 producing B cells (Breg) in multiple sclerosis is accompanied by a reduced naïve/memory Breg ratio during a relapse but not in remission. J Neuroimmunol.

[41] Disselhorst JA (2010). Image-quality assessment for several positron emitters using the NEMA NU 4-2008 standards in the Siemens Inveon small-animal PET scanner. J Nucl Med.

[42] Mattner F (2013). Central nervous system expression and PET imaging of the translocator protein in relapsing-remitting experimental autoimmune encephalomyelitis. J Nucl Med.

[43] Frost G (2014). The short-chain fatty acid acetate reduces appetite via a central homeostatic mechanism. Nat Commun.

[44] Solingapuram Sai KK (2017). Automated synthesis of 1-[11C]acetoacetate on a TRASIS AIO module. Appl Radiat Isot.

[45] Bakshi I (2018). Fructose bisphosphatase 2 overexpression increases glucose uptake in skeletal muscle. J Endocrinol.

[46] Small L (2018). Acute activation of pyruvate dehydrogenase increases glucose oxidation in muscle without changing glucose uptake. Am J Physiol Endocrinol Metab.

[47] Chambers MC (2012). A cross-platform toolkit for mass spectrometry and proteomics. Nat Biotechnol.

