# Supplementary material for: Gut-derived acetate promotes B10 cells with antiinflammatory effects
Source: JCI Insight. 2021 Apr 8;6(7):e144156. doi: 10.1172/jci.insight.144156 (PMC8119207; doi:10.1172/jci.insight.144156)
Supplement: Supplemental data [file jciinsight-6-144156-s204.pdf]

Supplemental Information

Gut-derived acetate promotes B10 cells with anti-inflammatory effects

C. I. Daïen , J. Tan, R. Audo, J. Mielle, L.E. Quek, J. R. Krycer, A. Angelatos, M. Duraes, G. Pinget, D. Ni, R. Robert, M.J. Alam, M. C. B. Amian, F. Sierro, A. Parmar, G. Perkins, S. Hoque, A. K Gosby, S.J. Simpson, R. Ribeiro, C. R Mackay, L. Macia

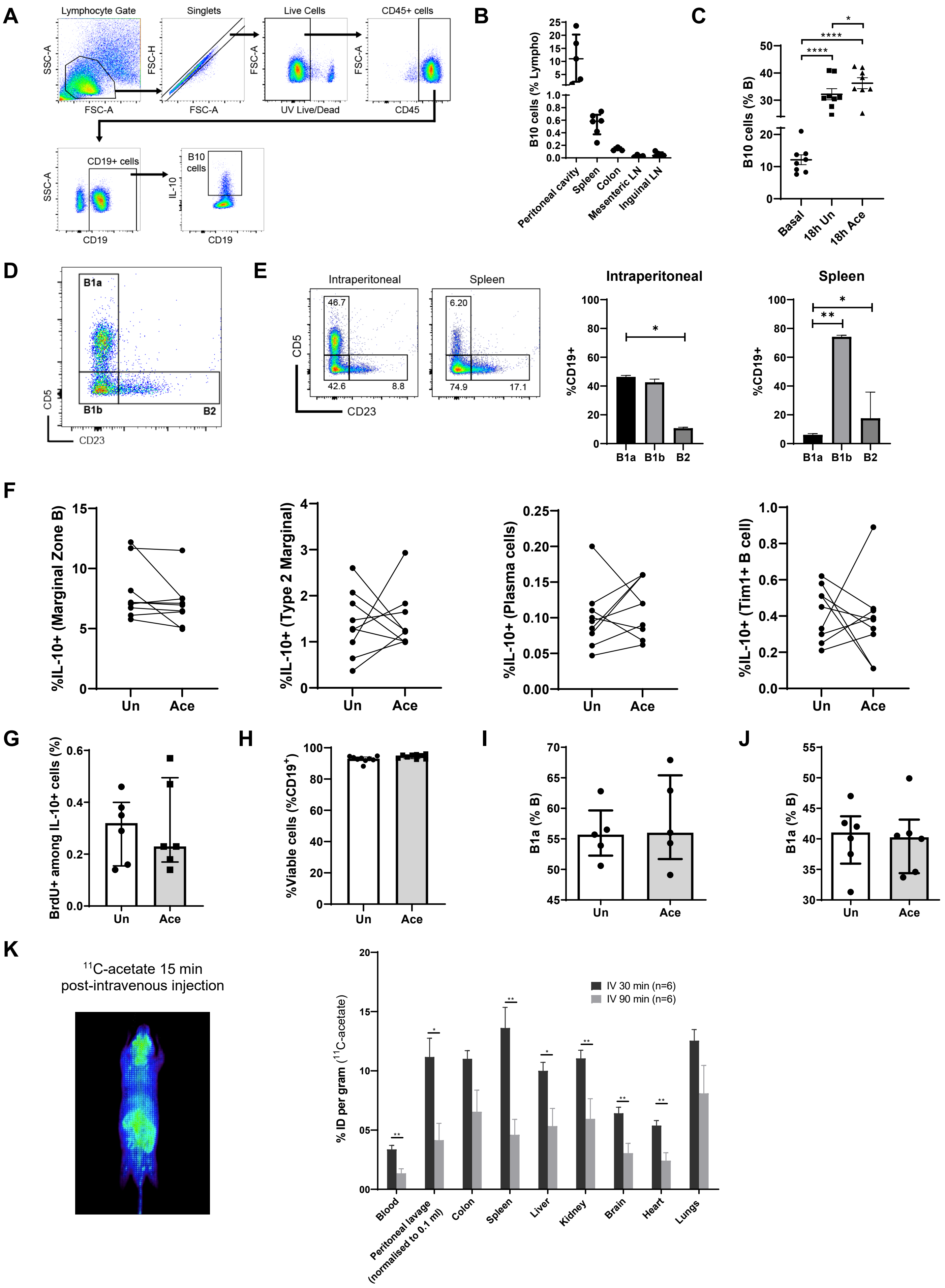

A
Lymphocyte Gate
Singlets
Live Cells
CD45+ cells
SSC-A
FSC-A
FSC-H
UV Live/Dead
CD45
CD19+ cells
B10 cells
IL-10
CD19
B
B10 cells (% Lympho)
Peritoneal cavity
Spleen
Colon
Mesenteric LN
Inguinal LN
C
B10 cells (% B)
Basal
18h Un
18h Ace
D
B1a
B1b
B2
CD5
CD23
E
Intraperitoneal
Spleen
46.7
42.6
8.8
6.20
74.9
17.1
%CD19+
B1a
B1b
B2
F
%IL-10+ (Marginal Zone B)
%IL-10+ (Type 2 Marginal)
%IL-10+ (Plasma cells)
%IL-10+ (Tim1+ B cell)
Un
Ace
G
BrdU+ among IL-10+ cells (%)
H
%Viable cells (%CD19+)
I
B1a (% B)
J
K
11C-acetate 15 min post-intravenous injection
% ID per gram (11C-acetate)
IV 30 min (n=6)
IV 90 min (n=6)
Blood
Peritoneal lavage (normalised to 0.1 ml)
Colon
Spleen
Liver
Kidney
Brain
Heart
Lungs

Supplementary Figure 1: Acetate promotes the differentiation of IL-10 producing B cells from mouse B1a precursors

(A) Gating strategy for the identification of B10 cells defined as IL-10$^+$CD19$^+$ B cells. (B) The levels of B10 cells were assessed in the peritoneal cavity, spleen, colon, mesenteric and inguinal lymph nodes (LN) following 4-hour stimulation with 1 µM CpG. (C) The levels of peritoneal B10 cells were assessed under basal conditions (Basal) or stimulated without (Un) or with acetate (Ace) overnight (n=8). (D) Gating strategy for the identification of CD5$^+$CD23$^-$ B1a cells, CD5$^-$CD23$^-$ B1b cells and CD5$^-$CD23$^+$ B2 cells, from CD19 gated cells. (E) Relative percentage of CD5$^+$CD23$^-$ B1a cells, CD5$^-$CD23$^-$ B1b cells and CD5$^-$CD23$^+$ B2 cells in the intraperitoneal cavity and the spleen. (F) The proportion of IL-10+ producing marginal zone, type 2 marginal, plasma and Tim1 expressing B cells were determined after overnight stimulation with or without acetate (n=8). (G) Proliferation of B10 cells during overnight stimulation with media or 10 mM acetate (Ace) were assessed by BrdU incorporation. (H) Proportion of viable cells were determined by Live/Dead gating of singlet CD19$^+$ lymphocyte cells after overnight stimulation with media or 10 mM acetate (Ace) (I). Percentage of peritoneal B1a cells among total peritoneal B cells were quantified by flow cytometry of mice administered intraperitoneally with PBS (n=10) or acetate (500 mg/kg, pH adjusted, n=10) or (J) acetate (200 mM, n=6) in drinking water for 3 weeks. Median (IQR) are shown. (K) PET scan image 15 min following intravenous administration of ^{11}C-acetate (left) and biodistribution of ^{11}C-acetate represented as intensity dose per gram (%ID per gram) after 30 or 90min post administration (right). Median (IQR) are represented with *$p<0.05$; **$p<0.01$.

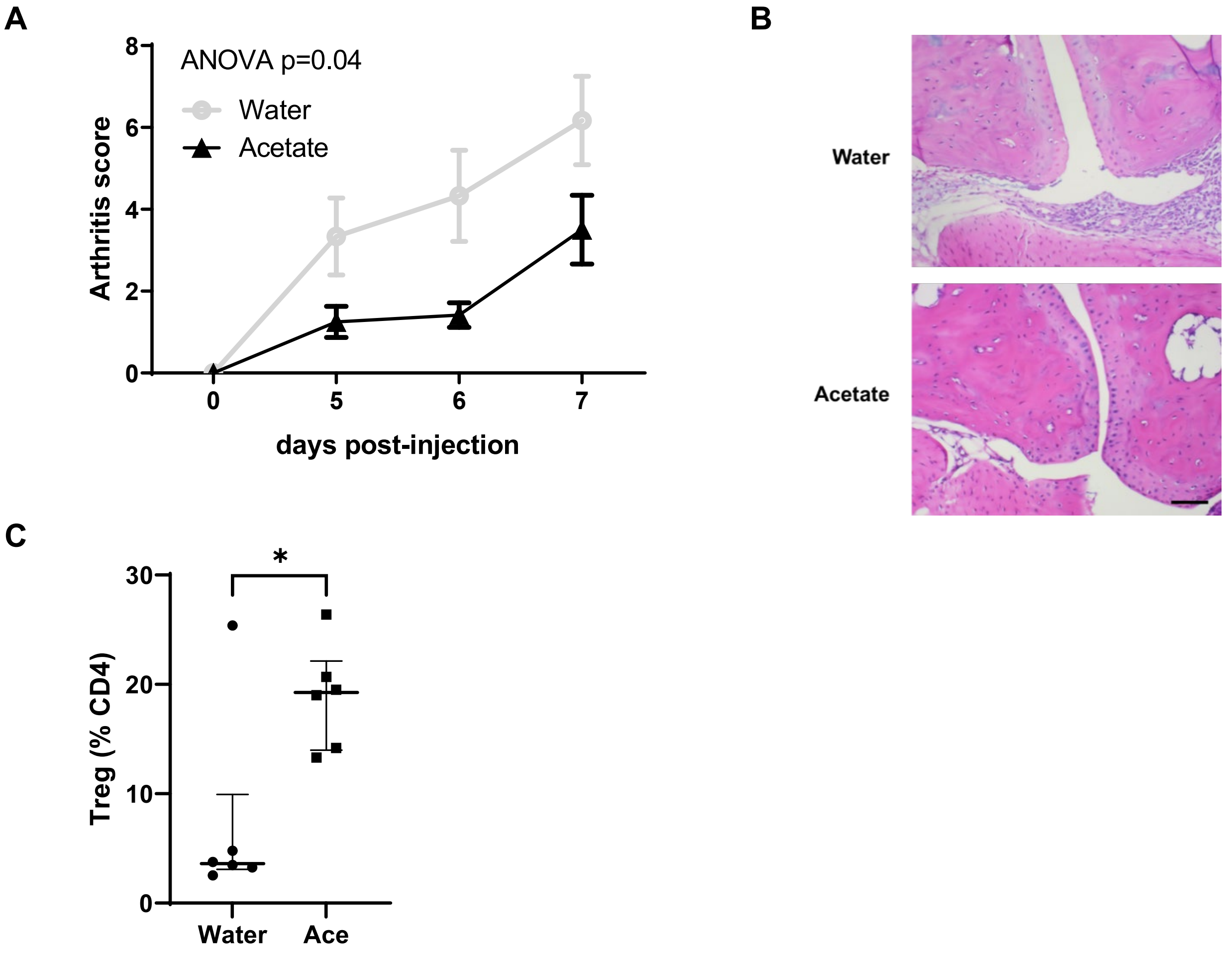

Supplementary Figure 2: Acetate decreases the severity of collagen-autoantibody-induced-arthritis

(A) Collagen antibody induced arthritis was induced by injecting i.p 4mg anti-collagen monoclonal antibodies at day 0 and 50 µg LPS at day 3. Water control (n=6) or 200mM acetate in drinking water (Acetate, n=6) was given to mice for 3 weeks before arthritis induction and during follow-up. Joints were monitored for 7 days. Two-way ANOVA was performed and data represented as mean (SEM). (B) Representative histological image of joints 7 days post arthritis induction. Scale bar represents 100µm (C) Proportion of splenic $CD4^{+}CD25^{+}FoxP3^{+}$ Treg cells were assessed by flow cytometry (n=6). Mann-Whitney tests was used for two-group comparison and data is represented as median (IQR). *p<0.05

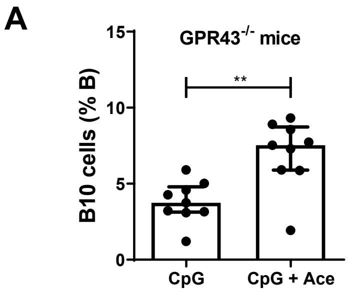

B

	range ~0-13000+						
Raw signal intensities	B1r1	B1r2	B1r3	B2r1	B2r2	B2r3	
	1.098635	1.846133	1.985014	43.08191	42.70527	38.56159	**Ffar2**
	20.3848	18.1201	23.8293	100.308	101.124	102.845	**Ffar1**
	0.604321	0.477723	0.611642	1.67766	2.17876	1.59568	**Ffar3**
	3754.32	3932.38	3643.06	4017.09	4152.77	4192.65	**Rpl13a**
relative to RPL13A	0.000293	0.000469	0.000545	0.010725	0.010284	0.009197	**Ffar2**
	0.00543	0.004608	0.006541	0.02497	0.024351	0.02453	**Ffar1**
	0.000161	0.000121	0.000168	0.000418	0.000525	0.000381	**Ffar3**

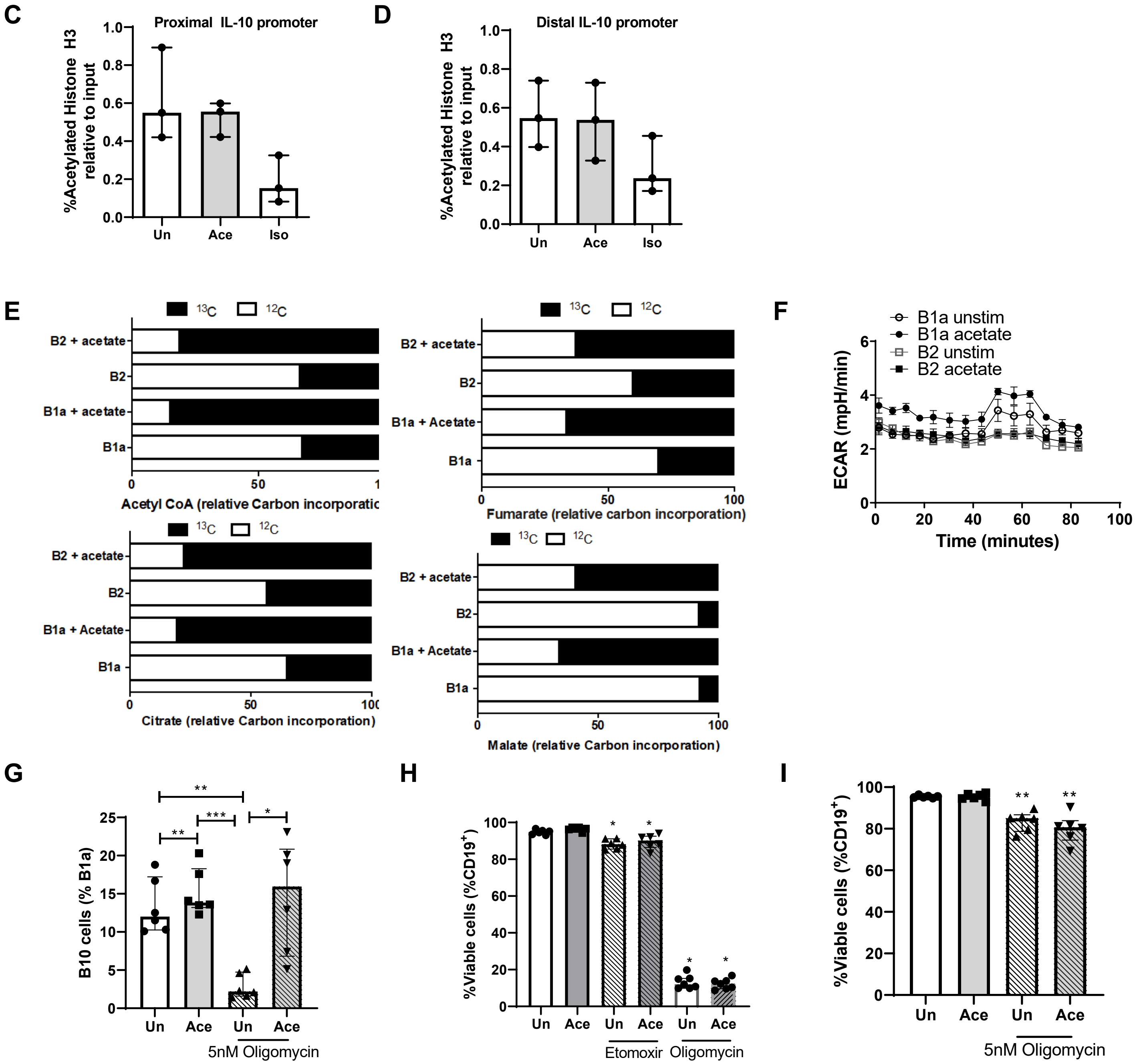

Supplementary Figure 3: Mechanisms behind the effects of SCFA on B10 cells

(A) 10^6 peritoneal cells isolated from *Gpr43*$^{-/-}$ mice (n=9) were cultured overnight with PBS (Un) or 10 mM acetate (Ace) with 1 µM CpG added during the last 4 hours of culture. Proportion of B10 cells (IL-

10^{+}CD19^{+} cells) was assessed by flow cytometry. (B) Relative gene expression of *Ffar1* (GPR40), *Ffar2* (GPR41) and *Ffar3* (GPR43) from open-source microarray data (GSE124827). (C-D) Percentage of acetylated-Histone H3 (Lys9) at the (C) IL-10 proximal and (D) distal promoter region relative to input control was determined from 10^6 peritoneal purified B1a cells incubated with either PBS (Un) or acetate alone (Ace, 10 mM) for 6 hours. Polyclonal rabbit IgG isotype control is also shown (Ig). (E) Relative incorporation of ^{13}C isolated B1a or B2 cells stimulated for 6 hours with 10mM ^{13}C acetate or PBS determined by liquid chromatography-mass spectrometry. (F) Real-time extracellular acidification rate (ECAR, mpH/min) was measured by seahorse assay from 0.5×10^6 sorted B1a or B2 cells stimulated overnight with our without acetate (10 mM) at baseline and after sequential injection of oligomycin (2 μM), FCCP (1 μM), Antimycin A + Rotenone (1 μM each). (G) 10^6 peritoneal cells were incubated overnight with 5 nM oligomycin in the presence or absence of 10 mM acetate; proportion of B10 cells among B1a cells quantified by flow cytometry (n=6). (H) Proportion of viable cells were determined by Live/Dead gating of singlet CD19^{+} lymphocyte cells after overnight stimulation with media or 10 mM acetate (Ace) in the presence of etomoxir or oligomycin (1μM). and (I) proportion of viable cells were determined by Live/Dead gating of singlet CD19^{+} lymphocyte cells (n=6). One-way ANOVA was performed and data represented as median (IQR) with *$p<0.05$, **$p<0.01$ and ***$p<0.005$.

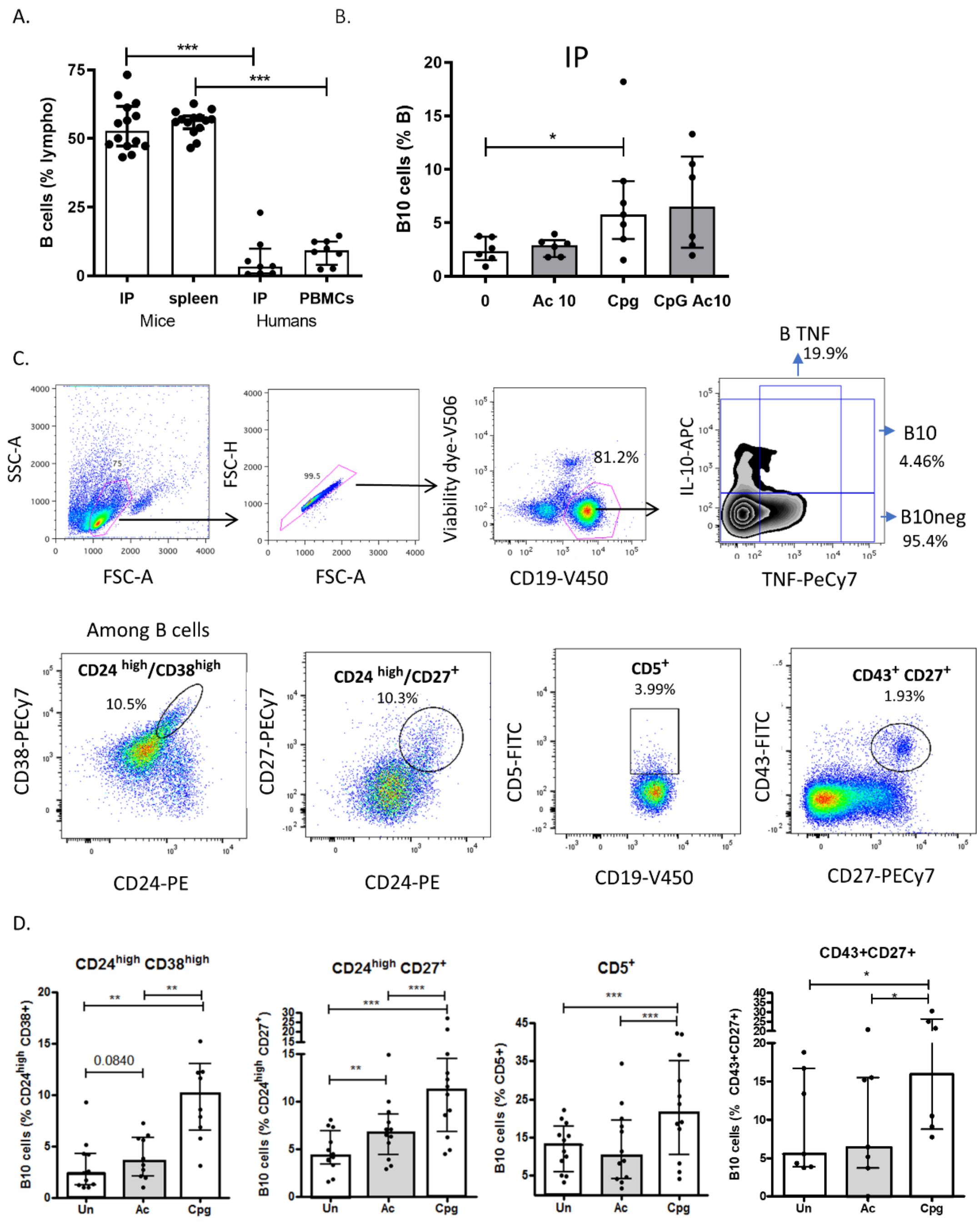

Supplementary Figure 4: Effect of acetate on human peritoneal cells.

Peritoneal cells of patients who underwent coelioscopy for exploration of ovarian cyst or peritoneal dialysis were harvested by peritoneal lavage. Human peritoneal cells were cultured overnight in presence of acetate (Ac 10 mM) without or with CpG, and activated with ionomycin, PMA for the last 4 hours in presence of Golgi-Plug. The levels of B cells were compared to human blood levels and to mouse peritoneal (IP) and splenic levels (A). Median (IQR) are shown, Mann-Whitney tests were used. *** p<0.001. In human peritoneal cells, B10 cells were assessed among B cells as IL-10$^+$CD19$^+$ cells

(n=6) (B). Median (IQR) are shown, Wilcoxson paired tests were used. * $p<0.05$.(C) Gating strategy of human B10 cells and their precursors. (D) B10 cells among human B10 precursors in presence of acetate (10 mM) or CpG (n=6-12). Median (IQR) are represented with *$p<0.05$, **$p<0.01$ and ***$p<0.005$.

Supplementary Material and Methods

Assessment of acetylated-Histone H3 at the IL-10 promoters

Determination of Histone H3 acetylation status at the IL-10 promoter regions was performed using the ChIP kit (Abcam) following the manufacturer's instruction. Briefly, 0.5 x 10^6 purified B1a cells were stimulated with or without 10mM acetate for 6 hours and then fixed with 1.1% paraformaldehyde (Biolegend) for 10min at room temperature. Chromatin was sheared using the Q800R2 Sonicator (Qsonica) – 30 cycles consisting of 15 seconds ON and 30 seconds OFF at 70% amplitude. Immunoprecipitation was performed using the Anti-acetyl-Histone H3 (Lys9) antibody (Merck; #07-352) or rabbit monoclonal IgG isotype control (DA1E; Cell Signaling) as negative control. Primers targeting the IL-10 proximal promoter region, sense 5'-GGAGGAGGAGCCTGAATAAC-3', anti-sense 5'-CTGTTCTTGGTCCCCCTTTT-3' and IL-10 distal promoter region, sense 5'-AACTCAGCCTGGAACTGACC-3', anti-sense 5'-GCCTCTCCTCCTGACACTCTT-3' were used as reported previously (1). Quantitative PCR was performed to determine the relative level of acetylation at target region relative to input DNA control.

Supplementary References

1. Bakshi I et al. Fructose bisphosphatase 2 overexpression increases glucose uptake in skeletal muscle. *J. Endocrinol.* 2018;237(2):101–111.
